# Supramolecular assembly of the beta-catenin destruction complex and the effect of Wnt signaling on its localization, molecular size, and activity in vivo

**DOI:** 10.1371/journal.pgen.1007339

**Published:** 2018-04-11

**Authors:** Kristina N. Schaefer, Teresa T. Bonello, Shiping Zhang, Clara E. Williams, David M. Roberts, Daniel J. McKay, Mark Peifer

**Affiliations:** 1 Curriculum in Genetics and Molecular Biology, University of North Carolina at Chapel Hill, Chapel Hill, NC, United States of America; 2 Department of Biology, University of North Carolina at Chapel Hill, Chapel Hill, NC, United States of America; 3 Department of Biology, Franklin and Marshall College, Lancaster, PA, United States of America; 4 Lineberger Comprehensive Cancer Center, University of North Carolina at Chapel Hill, Chapel Hill, NC, United States of America; University of Michigan, UNITED STATES

## Abstract

Wnt signaling provides a paradigm for cell-cell signals that regulate embryonic development and stem cell homeostasis and are inappropriately activated in cancers. The tumor suppressors APC and Axin form the core of the multiprotein destruction complex, which targets the Wnt-effector beta-catenin for phosphorylation, ubiquitination and destruction. Based on earlier work, we hypothesize that the destruction complex is a supramolecular entity that self-assembles by Axin and APC polymerization, and that regulating assembly and stability of the destruction complex underlie its function. We tested this hypothesis in *Drosophila* embryos, a premier model of Wnt signaling. Combining biochemistry, genetic tools to manipulate Axin and APC2 levels, advanced imaging and molecule counting, we defined destruction complex assembly, stoichiometry, and localization in vivo, and its downregulation in response to Wnt signaling. Our findings challenge and revise current models of destruction complex function. Endogenous Axin and APC2 proteins and their antagonist Dishevelled accumulate at roughly similar levels, suggesting competition for binding may be critical. By expressing Axin:GFP at near endogenous levels we found that in the absence of Wnt signals, Axin and APC2 co-assemble into large cytoplasmic complexes containing tens to hundreds of Axin proteins. Wnt signals trigger recruitment of these to the membrane, while cytoplasmic Axin levels increase, suggesting altered assembly/disassembly. Glycogen synthase kinase3 regulates destruction complex recruitment to the membrane and release of Armadillo/beta-catenin from the destruction complex. Manipulating Axin or APC2 levels had no effect on destruction complex activity when Wnt signals were absent, but, surprisingly, had opposite effects on the destruction complex when Wnt signals were present. Elevating Axin made the complex more resistant to inactivation, while elevating APC2 levels enhanced inactivation. Our data suggest both absolute levels and the ratio of these two core components affect destruction complex function, supporting models in which competition among Axin partners determines destruction complex activity.

## Author summary

Cell-cell communication is critical for cells to choose fates during embryonic development and often goes wrong in diseases like cancer. The Wnt cell signaling pathway provides a superb example. Loss of negative regulatory proteins like APC and Axin takes the brakes off cell proliferation and thus contributes to colon cancer. We study how APC, Axin and their partners keep cell signaling off, and how cell-to-cell Wnt signals reverse this. We use the fruit fly embryo, combining biochemical and genetic tools with advanced microscopy. We found that the destruction complex proteins APC2, Axin, and their antagonist Dishevelled are present at similar levels, allowing them to effectively compete with one another. We further find that the ability of Wnt signaling to turn off the negative regulatory destruction complex machine is influenced both by the levels of Axin and APC2 and by the ratio of their levels. We visualize the active destruction complex in the animal, and count the number of Axin proteins in this complex. Finally, we find that Wnt signals have two effects on the destruction complex—recruiting it to the plasma membrane and altering its assembly/disassembly. We then propose a new model for how this important signaling pathway is regulated.

## Introduction

Cell-cell signaling is critical for cell fate decisions during embryonic development and cell fate maintenance during adult homeostasis. Altered signaling by these same pathways underlies most solid tumors. The Wnt signaling pathway provides a paradigm—it regulates cell fate choice in tissues throughout the body, maintains stem cell identity in many adult tissues, and is inappropriately activated in colorectal and other cancers [1]. Thus, understanding the mechanisms by which signaling occurs and is regulated are key issues for cell, developmental, and cancer biology.

Work in both animal models and cultured mammalian cells provided a broad outline of Wnt signaling and its regulation [2,3]. The key effector is the transcriptional co-activator β-catenin (βcat; *Drosophila* Armadillo; Arm). In the absence of signaling, βcat is captured by a multiprotein complex called the destruction complex. The scaffold proteins Adenomatous polyposis coli (APC) and Axin bind βcat and present it to the kinases glycogen synthase kinase-3 (GSK3) and casein kinase 1 (CK1). They phosphorylate βcat, creating a binding site for an E3 ubiquitin ligase, thus targeting βcat for proteasomal destruction. When Wnt ligands bind to receptors, the destruction complex is downregulated, allowing βcat to accumulate, enter the nucleus and act together with the DNA binding proteins in the TCF/LEF family to transcriptionally activate Wnt-regulated genes.

Work in cultured mammalian cells has added important aspects to this model [2]—here we focus on the action of the destruction complex and its regulation by Wnt signaling. Several different mechanisms have emerged by which Wnt signaling downregulate βcat destruction and thus activate downstream signaling. Wnt binding to the Frizzled:LRP5/6 receptors triggers assembly of the receptors along with the Wnt effector Dishevelled (Dvl; *Drosophila* Dsh) into a higher order signalosome [4–7]. LRP5/6 becomes phosphorylated and recruits the destruction complex to the plasma membrane, at least in part by interactions between the phosphorylated tail of LRP5/6 and Axin[8]. The phosphorylated LRP5/6 tail can directly inhibit GSK3 [9]. Alternate mechanisms for destruction complex inhibition also exist. Dsh can co-polymerize with Axin via their shared DIX domains, antagonizing its function [10]. Careful kinetic analysis revealed that Wnt stimulation reduces the rate of ßcat phosphorylation by both CK1 and GSK3, reducing but not eliminating destruction complex activity [11]. Wnt signaling can trigger Axin dephosphorylation, reducing its interaction with both ßcat and LRP5/6, thus reducing ßcat destruction [12]. Finally, another study suggested that after Wnt signaling the destruction complex remains intact and capable of phosphorylating βcat, but its transfer to the E3 ligase is prevented [13]. These studies provide important insights into key regulatory mechanisms by which Wnt signaling can inactivate the destruction complex, but leave as an open question which mechanism(s) is most prominent during signaling in vivo.

The Wnt pathway is part of an emerging theme in cell signaling, in which self-assembly of multiprotein supramolecular signaling hubs creates non-membrane bound cellular compartments [14]. Three key steps in Wnt signaling are catalyzed by distinct supramolecular machines—the signalasome, involved in Wnt reception and destruction complex downregulation, the destruction complex itself, and the enhancesome, which mediates Wnt-regulated gene expression [3]. Key questions remain about the mechanism by which the active destruction complex targets ßcat for destruction in the absence of Wnt signaling. APC was originally viewed as the scaffold around which the destruction complex assembled, but subsequent work revealed that Axin fulfills this function, leaving APC’s molecular role a mystery. Further, while the destruction complex is typically represented in models as a simple four-protein complex, considerable evidence supports the idea that it is a large supramolecular protein assembly, built by self-polymerization of Axin and APC (e.g., [15–18]).

Recent work provided new mechanistic insights into the molecular mechanisms by which APC functions, helping begin to transform the static, low-resolution textbook model of Wnt signaling into a more dynamic, high resolution view. Super-resolution microscopy of Axin and APC complexes assembled after overexpression in colorectal cancer cells provided the first look inside the active destruction complex. Axin and APC containing “puncta” were resolved into intertwined strands of each protein, presumably assembled by polymerization [17]. Combining this with assessment of APC and Axin dynamics and genetic and biochemical dissection of the two proteins provided novel mechanistic insights and a new model. First, they suggest APC promotes/stabilizes Axin multimerization, thus increasing destruction complex efficiency [17]. Second, they revealed a key role for two peptide motifs in APC, 20 amino acid repeat 2 and sequence B/the CID, both essential for destruction complex function [19,20]. These motifs appear to play two roles. They are binding sites for alpha-catenin, stabilizing ßcat association with APC and preventing its dephosphorylation [21]. After Axin-mediated βcat phosphorylation, these APC motifs are also phosphorylated, triggering a regulated conformational change that transfers βcat out of the destruction complex to the E3 ligase, to restart the catalytic cycle [17]. These data fit with other studies suggesting that Wnt signaling does not totally turn off the destruction complex, but reduces the rate of destruction. Instead, the destruction complex remains intact and capable of phosphorylating βcat, but βCat transfer to the E3 ligase is inhibited [11,12,13].

However, this work was largely done in cultured cells, which provide a simple place to explore pathway circuitry but do not provide a physiologically relevant situation with all regulatory mechanisms intact. We thus took these insights back into the *Drosophila* embryonic epidermis, arguably the system where our understanding of the roles and regulation of the Wnt pathway is strongest. Stripes of cells in each body segment produce a fly Wnt, Wingless (Wg), creating a field of cells experiencing high, moderate and low levels of Wg signaling. Taking advantage of new genetic approaches and high-resolution microscopy, we addressed several key issues in the field, exploring the structure, assembly and stoichiometry of the destruction complex in vivo during normal development and how it is downregulated by Wnt signaling.

To understand a complex multiprotein machine, one key issue involves the relative levels of its component parts. Most current models of Wnt regulation suggest Axin accumulates at levels dramatically lower than those of other proteins in the destruction complex. This hypothesis derives from influential early work in *Xenopus* oocyte extracts. By adding in known amounts of recombinant Axin and measuring the resulting destruction complex activity, they estimated Axin concentrations were as much as 5000-fold lower than those of APC and other destruction complex proteins. Their mathematical model of Wnt signaling and many subsequent ones are based on these estimates [22–24]. In contrast, recent work in cultured mammalian cells suggests Axin and APC levels are more similar [25]. Thus, defining the relative levels of Axin and APC in tissues undergoing Wnt signaling in vivo is a key issue, and the *Drosophila* embryo provided a superb place to accomplish this end.

With relative protein levels defined, different models for the function and regulation of the destruction complex can be tested by varying absolute levels of Axin or APC and their relative ratios to one another. Substantially elevating Axin levels in *Drosophila* embryos strongly inhibits Wnt signaling [26]. Further analyses suggested there is a threshold below which elevating Axin does not substantially alter signaling, since more subtle elevation of Axin levels (2–5 fold) had little effect in *Drosophila* embryos or imaginal discs [27–29] and mutating *tankyrase*, which elevates Axin levels 2–3 fold, does not substantially perturb Wnt signaling [28,30]. In contrast, a 9-fold increase in Axin levels inhibited Wnt signaling in imaginal discs [27]. However, these studies used multiple tissues or systems in parallel, and left the mechanisms underlying the dose-sensitive response unclear. The *Drosophila* embryo provided a place to assess how altering Axin levels affects cell fate choice, Wnt-target gene expression and ßcat levels in parallel, and to directly compare effects on cells receiving and not receiving Wnt signals. It also offered the opportunity to manipulate APC levels, the other key scaffolding protein in the destruction complex. Whether APC levels are rate-limiting remains an open question, because APC has been viewed as present in substantial excess. The *Drosophila* embryo also allowed us to test effects of varying the Axin:APC ratio, another key parameter of any molecular model.

Finally, to effectively understand destruction complex assembly and function, we need to visualize it directly. Our recent super-resolution imaging of Axin:APC puncta in cultured cells provided the first insights into the internal structure and dynamics of this multiprotein machine, but these experiments involved significant over-expression. The *Drosophila* embryo provided a place to assess whether similar complexes assemble at near endogenous levels. Recent advances in molecular counting technology also offered the possibility of directly assessing the number of Axin proteins assembled in a complex.

Visualizing the destruction complex in the embryo would also allow us to address how Wnt signaling inactivates it. Work in cultured cells led to a model in which Wnt binding the Frizzled:LRP5/6 receptor complex triggers LRP5/6 phosphorylation, and Axin and Dvl/Dsh membrane recruitment [31]. What happens next is disputed, with many events suggested to play a part. For example, some data suggest the destruction complex is disassembled because Dsh competes for Axin [10] or Wnt signaling destabilizes Axin [32]. Interestingly, examining effects of Wg signaling on the destruction complex in *Drosophila* embryos led to starkly divergent conclusions. One group reported that Wg signaling strongly reduced Axin levels, as assessed both by immunofluorescence and immunoblotting [33]. A second, visualizing GFP-tagged Axin, found little or no effect of Wg on Axin levels—instead their data suggested that Wg signaling causes a Dsh-dependent relocalization of Axin from cytoplasmic puncta to the plasma membrane [26]. Finally, a third group reported that Wg signaling initially stabilizes Axin, as assessed by immunofluorescence, increasing both membrane bound and cytoplasmic pools [28,34]. Thus, the effects of Wg signaling on Axin, a key part of the mechanism underlying βcat stabilization, also remain an open question. Our system, allowing direct detection of fluorescently-tagged Axin expressed at near endogenous levels, allowed us to address this issue.

## Results

### *axin* and *APC1/APC2* are transcribed at similar levels

Most current models of Wnt regulation suggest Axin accumulates at levels dramatically lower than those of other destruction complex proteins, potentially making destruction complex activity sensitive to very small increases in its levels. However, the literature contains indications that this is not universally true (e.g. [25]). To better understand how APC and Axin levels affect Wnt signaling in vivo we directly compared levels of APC family members and Axin in *Drosophila* embryos.

**Fig 1:**
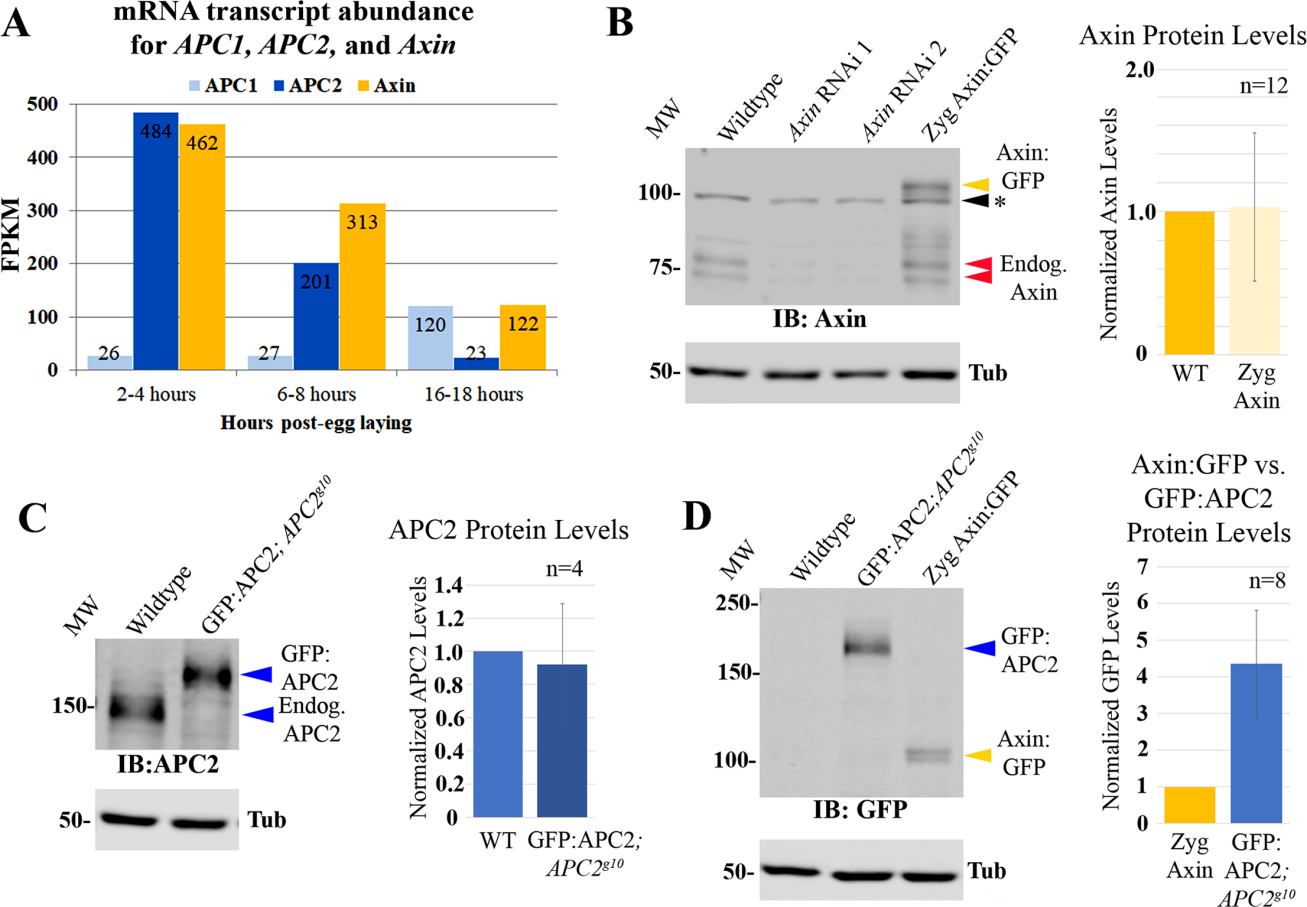
Endogenous APC2 and Axin proteins accumulate at similar levels. (A) mRNA levels (RNAseq) of *APC1* (light blue), *APC2* (blue), and *Axin* (yellow) during *Drosophila* embryogenesis. Levels are Fragments Per Kilobase of transcript per Million mapped reads (FPKM). (B-D) Immunoblots, 4-8hr old *Drosophila* embryos. Tubulin is loading control. n = # of blots quantified (S1 Table). (B) Anti-Axin antibody. Endogenous Axin levels versus those in Axin RNAi or Zyg Axin:GFP embryos. Endogenous Axin runs as doublet ~75kDa (red arrowheads) while Axin:GFP runs at ~105kDa (yellow arrowhead). * = background band. (C) Anti-APC2 antibody. Endogenous APC2 levels versus those of a GFP:APC2 transgene expressed under its endogenous promoter in an *APC2* null (*APC2*^*g10*^) background. (D) Anti-GFP antibody. Relative levels of GFP:APC2 expressed under its endogenous promoter versus Zyg Axin:GFP.

We first compared mRNA levels of *Drosophila axin* with those encoding the two fly APC family proteins, *APC1* and *APC2*, using RNAseq data from staged embryos. In embryos, APC2 plays the predominant role in Wnt regulation during early to mid-embryogenesis ([35–37]; 2–4 or 6–8 hours after egg laying, respectively), while APC1 is expressed at low levels early but becomes prominent later in the central nervous system [38,39]. Consistent with this, *APC2* mRNA levels are ~19x higher than *APC1* during early embryogenesis, and ~7x higher during mid-embryogenesis (484 versus 26 Fragments Per Kilobase of transcript per Million mapped reads (FPKM), and 201 versus 27 FPKM, respectively). However, in late embryogenesis, as the nervous system is assembled, *APC1* mRNA levels are ~5x more abundant than APC2 (120 vs. 23 FPKM). Since APC2 and APC1 can act redundantly in regulating Wnt signaling [36,37], we compared *axin* mRNA levels with combined mRNA abundance of *APC1* plus *APC2*. Surprisingly, RNAseq reads for *axin* were roughly comparable to those of *APC1* plus *APC2* at three different stages of embryonic development (Fig 1A), indicating that there are not dramatic differences between APC family members versus Axin at the mRNA level.

### Axin and APC2 proteins accumulate at similar levels during early-mid embryogenesis

These data did not rule out differences in protein translation or stability. To determine if similar mRNA levels led to similar protein levels, we compared Axin and APC2 protein levels in early to mid-embryogenesis (4–8 hrs), when APC2 is the predominant family member expressed. Since antibodies to APC2 and Axin may have different affinities, one cannot simply compare antibody-labeled endogenous proteins. To overcome this, we utilized GFP-tagged proteins expressed at near-endogenous levels. This allowed us to compare endogenous versus GFP-tagged Axin, or endogenous versus GFP-tagged APC2 proteins, using antibodies against the endogenous proteins, followed by comparing GFP-tagged Axin and GFP-tagged APC2 proteins, using anti-GFP antibodies. We used the GAL4-UAS system [40,41] to express Axin:GFP, using the driver that gave the lowest level of Axin:GFP expression (*act5c*-GAL4 provided by male parents). Axin:GFP was expressed at 1.0±0.5 fold that of endogenous Axin, as assessed by immunoblotting with anti-Axin antibodies (Fig 1B, S1 Table). We next used transgenic flies expressing GFP:APC2 under control of the endogenous *APC2* promotor, in an *APC2* null mutant background [19]. Using anti-APC2 antibodies, we re-confirmed that *APC2*-driven GFP:APC2 was expressed at the same level as endogenous APC2 (0.9±0.4 fold endogenous APC2; Fig 1C, S1 Table). To complete the comparison, we then compared *APC2*-driven GFP:APC2 to Axin:GFP driven by zygotic *act5c*-GAL4. Immunoblotting with anti-GFP antibodies revealed that GFP:APC2 is expressed ~4-fold the levels of Axin:GFP (Fig 1D; 4.3±1.4; S1 Table). These three comparisons—endogenous Axin to *act5c*-GAL4 driven Axin:GFP, *act5c*-GAL4 x Axin:GFP to *APC2-*driven GFP:APC2, and *APC2-*driven GFP:APC2 to endogenous APC2—provided a reasonable estimate of the relative levels of endogenous APC2 to Axin: APC2 accumulates at a ~5-fold higher level than Axin (4.7±1.4). This is in contrast to the 5000-fold difference in accumulation observed in *Xenopus* extracts that forms the basis of some current models, but is consistent with the similar levels of mRNAs revealed by RNAseq.

### Developing methods to vary Axin levels during embryogenesis

Axin is the key scaffold on which the destruction complex is built, and thus most models of Wnt signaling suggest Axin is rate limiting for destruction complex function. Previous experiments in fly embryos and imaginal discs strongly support this, as over-expressing Axin can shut down Wnt signaling. Our knowledge of the relative levels of APC2 versus Axin in the *Drosophila* embryonic epidermis allowed us to confirm and extend the analysis. We first developed ways to vary Axin levels systematically, exploring how increasing Axin levels to different degrees altered viability, cell fate and expression of a Wg target gene. We next explored the underlying mechanism, by examining how different Axin levels affected destruction complex activity and ßcat levels, both in cells receiving and not receiving Wg signals. We then brought APC2 into this picture, examining effects of elevating APC2 levels, and of altering the ratios of Axin to APC2.

To manipulate Axin levels systematically, we used the GAL4-UAS system. Four crosses using two different GAL4 drivers provided different levels and timing of Axin over-expression (S1 Fig; Methods; S1 Table). *act5c*-GAL4 is expressed during oogenesis and relatively ubiquitously during embryonic development. 1. By crossing UAS-Axin:GFP females to *act5c*-GAL4/+ males, we achieved lower-level and later elevation of Axin:GFP levels, which was driven by zygotically-expressed GAL4 (hereafter Zyg Axin). 2. By crossing *act5c*-GAL4/+ females to UAS-Axin:GFP males (hereafter Mat/Zyg Axin), we achieved relatively high-level overexpression, which began early due to maternally-contributed GAL4 and continued zygotically. The second GAL4 driver stock was MatGAL4, which includes two GAL4 lines expressed during oogenesis; they are not expressed zygotically but maternally expressed GAL4 protein perdures in the embryo. 3. For maternal and zygotic over-expression, we assessed progeny of females trans-heterozygous for MatGAL4 and UAS-Axin:GFP (hereafter Mat Axin). 4. To achieve levels of Axin elevation intermediate between that produced by Zyg Axin and Mat Axin, we used MatGAL4 to co-express UAS-Axin:GFP with a second UAS-driven transgene encoding RFP (hereafter Mat RFP&Axin). When two different UAS-driven transgenes are present, this reduces expression of both transgenes. We directly measured protein levels by immunoblotting with antibodies to either GFP or to endogenous Axin.

**Fig 2:**
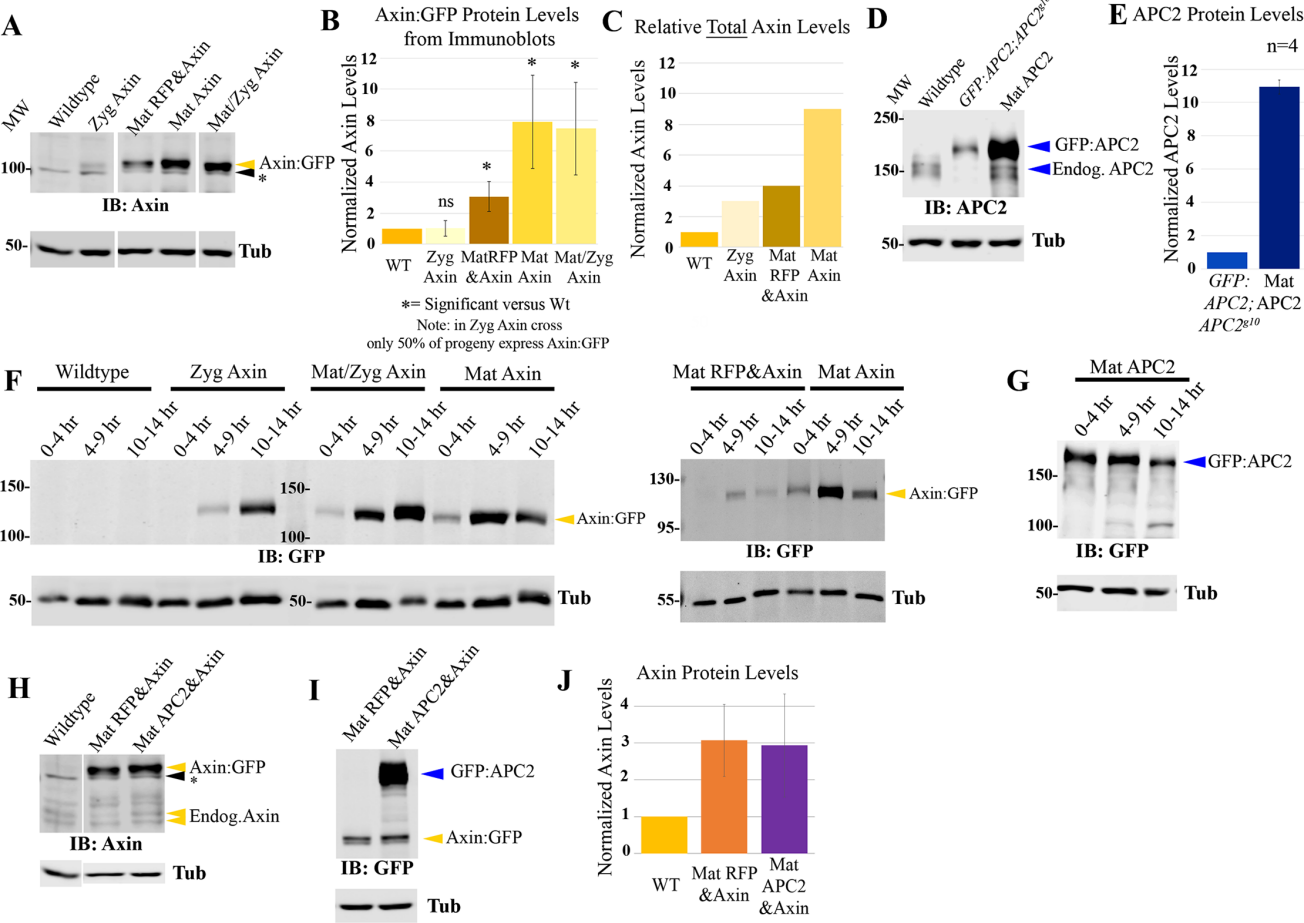
Developing tools to differentially elevate levels of Axin:GFP. (A-E) Immunoblots and quantification, 4-8hr old *Drosophila* embryos. Tubulin is loading control. (A) Anti-Axin antibody—samples were run on same blot with intervening lanes removed. Levels of Axin:GFP when expressed with different GAL4 drivers. * = background band. (B) Quantification of Axin:GFP, normalized to levels of endogenous Axin. # of blots quantified is in S1 Table. (C) Relative levels of total Axin (thus including both endogenous Axin + Axin:GFP) accumulation—see S1 Table for standard deviation and # of blots assessed. (D,E) Anti-APC2 antibody. Endogenous APC2, GFP:APC2 expressed via its endogenous promoter, or GFP:APC2 expressed using MatGAL4. (F,G) Immunoblots of *Drosophila* embryos of the indicated ages, anti-GFP Antibody. Time courses of Axin:GFP (F) or GFP:APC2 (G) accumulation when expressed with different GAL4 drivers. (H) Immunoblot of 4-8hr old *Drosophila* embryos, with anti-Axin antibody, comparing endogenous Axin levels to Axin:GFP levels in lines expressing both Axin:GFP and a second transgene (RFP or GFP:APC2). From same gel with intervening lanes removed. (I) Same samples stained with an anti-GFP antibody, thus comparing levels of Axin:GFP and GFP:APC2. (J) Quantification of Axin:GFP levels normalized to wildtype. N = 9 blots.

These four schemes produced an excellent range of Axin expression levels in stage 9 embryos, when Wnt signaling is at its peak. Zyg Axin effectively tripled normal Axin levels in embryos in which it was expressed (Fig 2A–2C, S1 Table; taking into account endogenous Axin and the fact that only 50% of embryos inherit the GAL4 driver). Mat RFP&Axin led to an ~4-fold increase, while both Mat/Zyg Axin and Mat Axin led to 8–9 fold elevation in total Axin levels (Fig 2A–2C, S1 Table). When we examined the pattern of Axin:GFP accumulation, we noted Mat/Zyg Axin led to substantially more variable expression from cell-cell than MatGAL4-driven Axin:GFP. Thus, in most subsequent functional assays we used Mat Axin for high-level overexpression. In addition to differences in expression levels, these lines also differed in timing of Axin:GFP expression (Fig 2F). Zyg Axin levels started very low (as expected with no maternal GAL4 expression) and continued to rise throughout development. Mat Axin levels started somewhat higher (driven by maternal GAL4), increased during stages 9–11 (4–9 hrs) and then slowly decayed. Mat/Zyg Axin exhibited initially modest Axin:GFP levels, which continued to rise throughout development. Mat RFP&Axin accumulation followed a similar expression pattern as Mat Axin, but at decreased levels due to the presence of two UAS-driven transgenes (Fig 2F, right). These tools allowed us to vary Axin levels systematically, and we thus used them to assess how altering Axin levels and timing of accumulation affect Wg signaling and its regulation, by assessing effects on embryonic viability, cell fate choice, Wg target gene expression, and Arm (fly βcat) levels.

### When Axin expression is elevated by ≥4-fold, it inhibits Wg-regulated cell fate choice during embryogenesis

**Fig 3:**
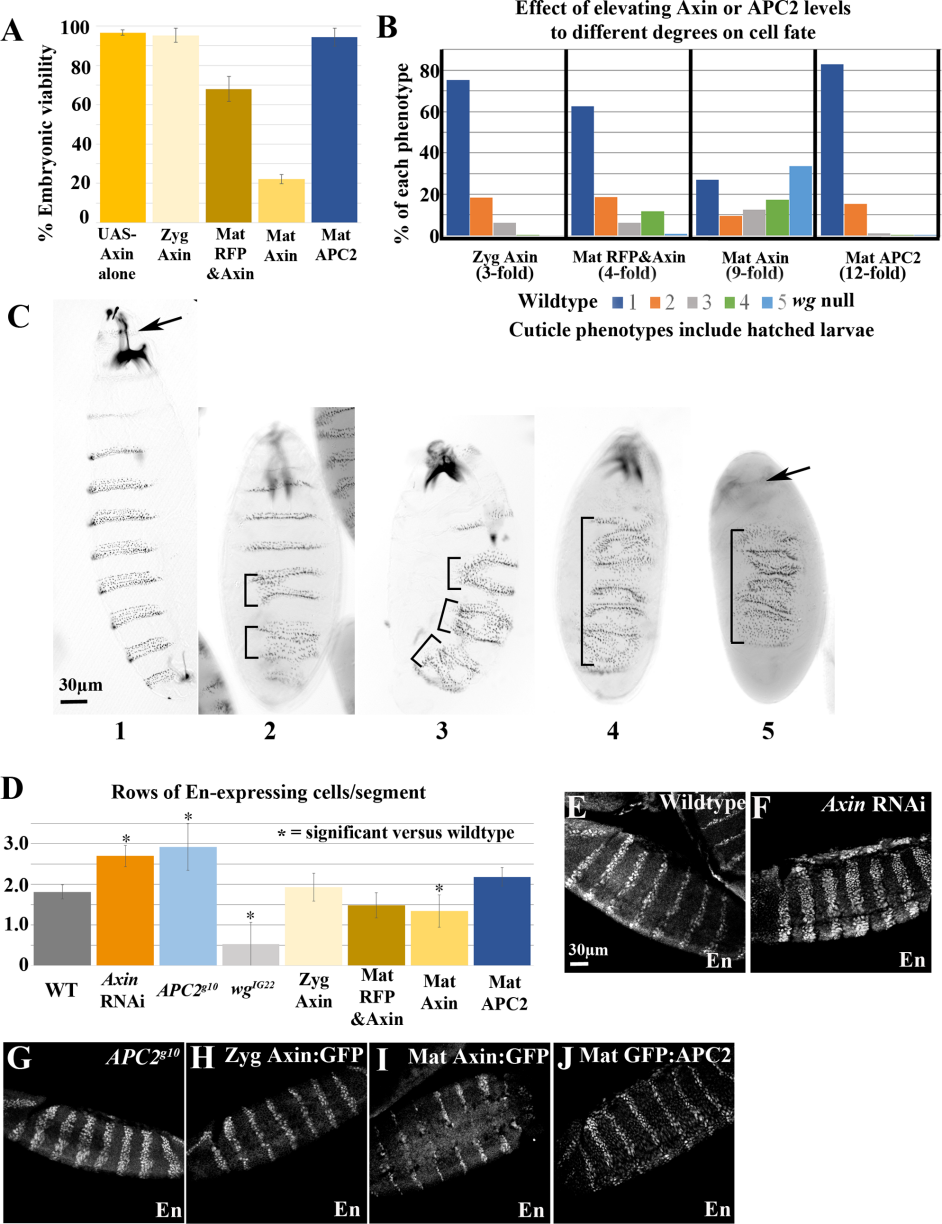
Elevating Axin produces dose-sensitive inhibition of Wg signaling, while increasing APC2 levels does not. (A) Embryonic viability of indicated genotypes. (B,C) Assessing the effect of elevating Axin or APC2 levels on Wg-regulated cell fates. (B) Range of cuticle phenotypes of embryos/larvae of each genotype—since not all genotypes are lethal, phenotypes include those of hatched larvae. (C) Representative images of cuticle phenotypes used in B. Anterior to the top. 1: Wildtype. 2: 1–2 merged denticle belts (brackets). 3: 3–4 merged denticle belts. 4: Most denticle belts merged, mouth parts still present. 5: *wg* null phenotype–denticle lawn and no head (arrow). (D) Quantification of number of rows of En-expressing cells per segment. Embryos analyzed: WT-21, AxinRNAi-5, APC2^g10^- 5, wg^IG22^–14, Zyg Axin- 9, Mat RFP&Axin- 11, Mat Axin- 18, Mat APC2- 12. * = p<0.05 using a one-way ANOVA test. (E-J) Representative images, En expression, as quantified in D. Anterior to the left.

We first assessed effects of elevating Axin levels on embryonic viability and cell fate choice—these assays integrate effects on Wnt signaling across embryonic development, and thus have to be interpreted in light of effects on Axin levels both at stage 9 and later, as Wg signaling affects cell fate choice through stage 11 (9 hours; [42]). The relatively subtle (3-fold) Axin elevation produced by Zyg Axin at stages 9–11 did not result in embryonic lethality (Fig 3A, S2 Table; 5% lethality vs. 3% lethality of wildtype controls (controls carried UAS-Axin without a GAL4 driver)). We then examined larval cuticles to look for more subtle effects on Wg signaling. Reducing Wg signaling affects cell fate, causing loss of naked cuticle fates and merger of denticle belts—Fig 3C illustrates the graded series of defects with successively reduced Wg signaling. Most Zyg Axin embryonic cuticles (3-fold increase) were near wildtype (Fig 3B and 3C, S3 Table), though the occasional defects seen suggest subtle reduction of Wg signaling in some embryos. Consistent with this possibility, no hatching Zyg Axin larvae survived to adulthood—this may reflect the fact that as a consequence of zygotic GAL4 expression Axin levels continued to rise throughout development (Fig 1F). The slightly higher level expression of Axin:GFP in Mat RFP&Axin embryos (4-fold increase) and the earlier onset of expression led to some embryonic lethality (32% lethal; Fig 3A; S2 Table), and a larger fraction of embryos had moderate inhibition of Wg signaling, as assessed by cell fate choices (Fig 3B and 3C, S3 Table). In contrast, higher-level, earlier overexpression of Axin (8–9 fold) led to substantial embryonic lethality—90% lethality for Mat/Zyg Axin and 78% lethality for Mat Axin (Fig 3A; S2 Table). In both crosses, there were two genotypes of embryonic progeny; for Mat/Zyg Axin these differed by whether or not they had a zygotic copy of *act5c*-GAL4 and for Mat Axin by whether they had one or two copies of the UAS-Axin:GFP transgene zygotically (S1 Fig). Cuticle analysis of cell fates revealed that Wg-signaling was strongly reduced in many Mat/Zyg Axin and Mat Axin progeny (Fig 3B and 3C, S3 Table), but there were variations in the strength of this effect that likely reflect the two different zygotic genotypes in each cross. Thus, when levels of Axin exceed ~4–5 fold endogenous during stages 9–11, this led to embryonic lethality and strong inhibition of Wg-regulated cell fates.

To assess effects of Axin levels on a Wg-regulated target gene, we examined *engrailed* (*en*) expression, using antibodies to its protein product. En usually accumulates in the two most posterior cell rows in each body segment (Fig 3D and 3E, S4 Table), and maintenance of En expression requires Wg signaling—thus in *wg* mutants En stripes are narrowed ([42]; Fig 3D, S4 Table). In contrast, in *APC2*^*g10*^ null mutants or after Axin RNAi, En expression expands to additional cell rows (Fig 3D, 3F and 3G, S4 Table). The 3-fold elevation of Axin levels via ZygGAL4 did not affect En expression (Fig 3D and 3H, S4 Table). In contrast, the 9-fold increase of Axin via MatGAL4 led to partial loss of En expression (Fig 3D and 3I, S4 Table), though on average this was not as severe as that seen in *wg* mutants. Thus, mildly elevating Axin levels during the critical period (stage 9–11) has little effect on embryonic viability, Wg regulated cell fates or target genes, but when Axin levels are elevated ≥ 8-fold, Wg signaling is strongly inhibited, consistent with previous data suggesting that Axin is rate-limiting.

### Elevating Axin levels has no effect on Arm levels in cells not receiving Wg signals, but does render the destruction complex more resistant to inactivation by physiological levels of Wg signaling

**Fig 4:**
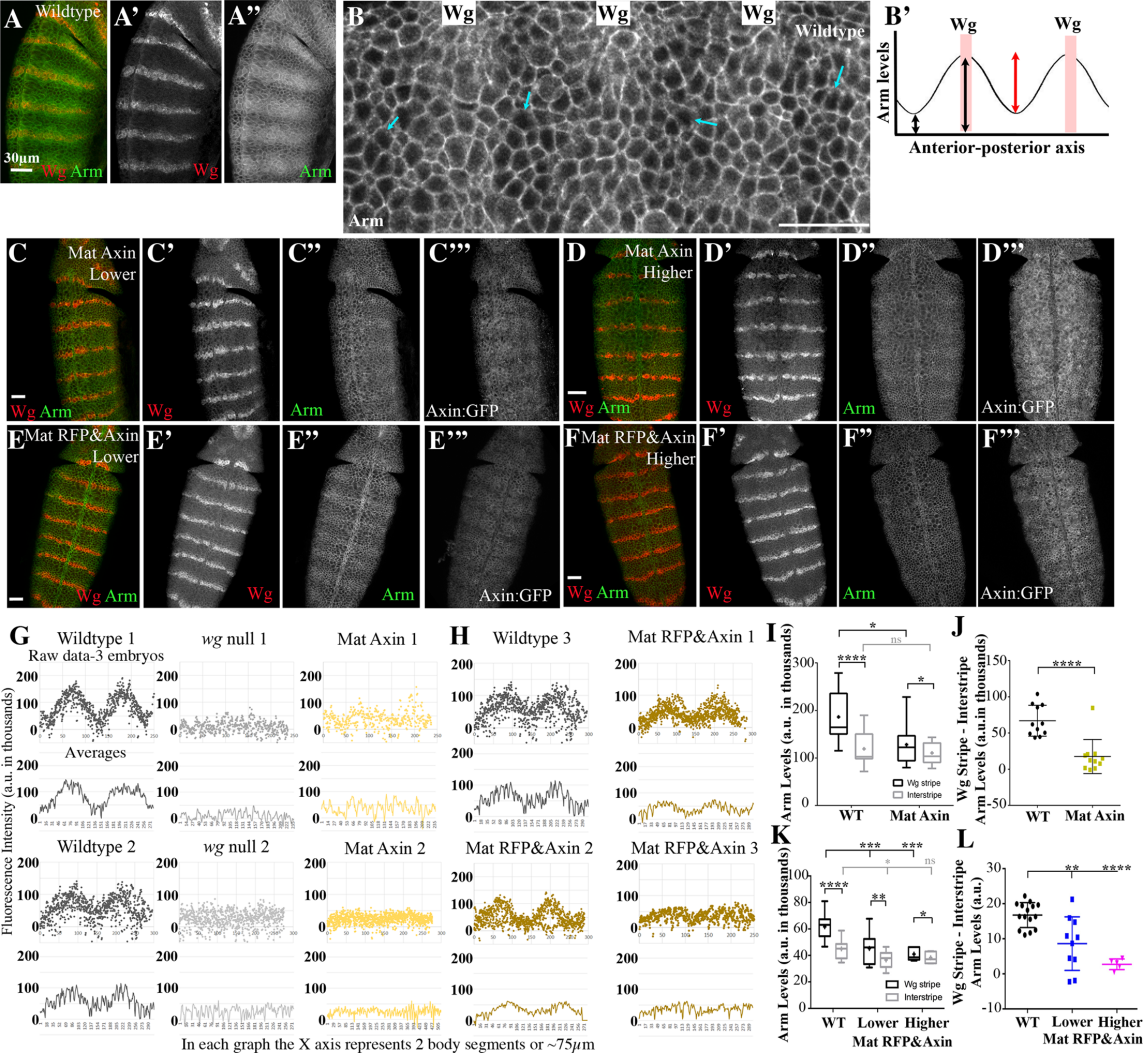
Increasing Axin levels reduces the ability of endogenous Wg signaling to turn down the destruction complex but has little or no effect in Wg-Off cells. (A-F) Fixed stage 9 embryos. Anterior to the top except B where anterior is to the right. (A,B) Representative images of Arm accumulation in wildtype embryos. (B) Close-up. “Wg” shows location of Wg-expressing cells, which accumulate elevated levels of Arm in the nucleus and cytoplasm. Arrows illustrate that in Wg-OFF cells, there are still detectable levels of Arm in the cytoplasm and also illustrate cytoplasmic retention. (B’) Diagrammatic illustration of Arm levels across two embryonic segments, illustrating the graded nature of Arm accumulation, and the parameters we assessed: absolute levels in Wg stripes or in interstripes (black arrows), and the difference between these levels (red arrow). (C,D) Representative images, Mat Axin:GFP embryos with higher or lower levels of Axin:GFP expression (depending on zygotic copy number of UAS- construct), taken from the same slide under the same microscope settings. Arm accumulation in Wg–stripes is reduced. (E,F) Representative images, Mat RFP&Axin embryos with higher or lower levels of Axin:GFP expression, taken from the same slide under the same microscope settings. Arm accumulation in Wg–stripes is reduced at higher levels of Axin:GFP expression, but less affected at lower levels. (G,H) When Axin levels exceed 4xendogenous, this flattens the usual graded level of Arm accumulation across each embryonic segment. Plots of Arm accumulation over 2 segments for each genotype indicated. Dot plots = raw data from 3 separate embryos. Line graphs underneath = averages of these data. (G) Left. In wildtype embryos Arm accumulation varies smoothly over the segment. Middle. Loss of Wg flattens the Arm stripes. Right. Expressing Axin:GFP using the MatGAL4 driver blunts or eliminates Arm stripes. (H) The slightly lower levels of Axin expression in Mat RFP&Axin embryos have more variable effects on Arm stripes. (I-L) Elevating Axin levels reduces Arm accumulation in Wg stripes but does not significantly affect Arm levels in interstripes. (I,K) Box and whisker plot comparing Arm accumulation levels in Wg-expressing stripes versus Arm levels in the interstripes for the indicated genotypes. n = 10 pairs. Boxes extend from 25^th^ to 75^th^ percentiles, and whiskers indicate minimum to maximum values. Median = middle line of the box and mean = +. (Full values are in S5 Table). (J,L) Scatter plots showing difference in Arm accumulation between the Wg stripes and interstripes within individual embryos. Each point = a single embryo. Error bars = mean+S.D. Statistical analysis: A paired t-test was used to determine the significance between intragroup values in I and K. To assess the significance between intergroup values, an unpaired t-test was used in I and J, and an ordinary one-way ANOVA followed by Dunnett's multiple comparisons test was applied in K and L. ns, not significant i.e. p**≥** 0.05. * = p<0.05. ** = p<0.01. *** = p<0.001. **** = p<0.0001. Scale bars = 30μm.

The primary role of the Axin/APC2 based destruction complex is to regulate levels of Arm/βcat. We thus measured effects of different Axin levels on Arm accumulation. Arm has two roles: as part of the cadherin-based cell adhesion complex and as a transcriptional co-activator in the Wnt pathway. Thus, all cells have a pool of Arm at the cortex in adherens junctions. In wildtype, Wg is expressed by one row of cells in each segment, and moves to neighboring cells, resulting in a gradient of Wg signaling across the segment. In cells not receiving Wg, the destruction complex binds to newly synthesized Arm, which targets it for destruction (Fig 4A and 4B). Thus, levels of cytoplasmic Arm are low. However, they are not zero; instead Arm that is not immediately destroyed is retained in the cytoplasm by binding to the multiple Arm binding sites on APC2 [19]. Together cytoplasmic retention and destruction mean little or no Arm can translocate to the nucleus and co-activate Wnt target genes (Fig 4B, arrows). In cells receiving Wg, the destruction complex is turned down, and Arm accumulates in both the cytoplasm and nucleus, leading to activation of Wnt target genes [43]. Together, these inputs create a gradient of Arm accumulation across the segment, with the highest level of cytoplasmic/nuclear Arm accumulation in Wg-expressing cells and their immediate neighbors, and gradually decreasing levels of cytoplasmic/nuclear accumulation in cells more distant from the Wg source (Fig 4A and 4B; diagrammed in 4B’). In *wg* mutants the destruction complex downregulates Arm in all cells, eliminating the stripes of Arm accumulation [43]. Because different GAL4 drivers changed both the level and the timing of Axin expression, we focused our attention on stage 9, when Wnt signaling is maximal, to alleviate the complication of differences in timing.

We developed methods to quantify the effects of elevating Axin levels on two different aspects of Arm stabilization. To quantify the graded effects of Wg signal across the segments (Fig 4B’), we used a digital image mask (S2A’ Fig) to remove the cortical Arm in cell-cell adherens junctions (S2A vs. S2A” Fig), and then measured fluorescence levels of cytoplasmic/nuclear Arm pixel by pixel across two to three body segments (S2A” Fig box; two wildtype examples are in Fig 4G left). In wildtype embryos, both our images and quantitative analysis revealed a smooth gradation of Arm accumulation, from peaks centered on Wg stripes to troughs in the interstripes (Fig 4A, 4B and 4G). As a control, we examined *wg* null mutants, in which Arm levels were not elevated in any cells (Fig 4G center; each mutant was analyzed in parallel with the wildtype shown to its left). 9-fold elevation of Axin (Mat Axin) led to either complete loss of this graded stabilization of Arm in cells receiving Wg signal, or a reduction in the height of the peaks, relative to wildtype (Fig 4C and 4D, quantified in G). The changes in Arm peak heights were dependent on the level of Axin:GFP expression; this was best visualized in Mat RFP&Axin embryos where the lower level Axin expression only partially flattened the Arm distribution (Fig 4E and 4F, quantified in 4H).

To measure absolute levels of Arm stabilization by Wg signaling, we assessed Arm fluorescence in two groups of cells: 1–2 cell rows centered on cells expressing Wg (the Wg stripes; S2B and S2B’ Fig, yellow boxes) and 1–2 cell rows farthest from the Wg-expressing cells (the interstripes; S2B Fig, white boxes). Wildtype embryos were included on the same slides as a control. We quantified absolute Arm levels in both Wg stripes and interstripes (Fig 4B’, black arrows, Fig 4I, S5 Table) and also the difference in levels between these two cell types (Fig 4B’ red arrow, Fig 4J, S6 Table). 9-fold overexpression of Axin (Mat Axin) substantially reduced Arm accumulation in Wg stripes, to levels similar to those normally seen in interstripes (Fig 4C and 4D vs. 4A; quantified in 4I and 4J, S5 and S6 Tables). However, strikingly, Arm accumulation in interstripes was unaffected. The 4-fold Axin overexpression in Mat RFP&Axin embryos also reduced Wg-stabilization of Arm, but when we sorted embryos by level of Axin:GFP expression, this was less pronounced in embryos with lower levels of Axin:GFP (Fig 4E and 4F vs. 4A, quantified in 4K and 4L, S5 and S6 Tables). To complete this analysis, we examined whether elevating Axin levels affected only the signaling pool of Arm (cytoplasmic plus nuclear) or also affected the pool at cell junctions. Using a membrane-mask, we separately assessed these two pools. Elevating Axin levels 9-fold (Mat Axin) reduced Arm accumulation in both the junctional and cytoplasmic/nuclear pool in Wg-ON cells, without significantly affecting either pool in Wg-OFF cells, relative to wildtype embryos (S3A–S3C Fig, S7 Table).

Together, these data suggest that when Axin levels are elevated ≥4–5 fold, the destruction complex cannot be effectively inactivated by physiological levels of Wg signaling, confirming previous observations that Axin is rate-limiting in this regard. However, it was also striking that elevating Axin levels did not further increase Arm destruction in cells not receiving Wg signal (Fig 4I, S5 Table, S3A–S3C Fig. S7 Table), suggesting that Axin is not rate-limiting for destruction complex activity in those cells.

### Levels of APC2 can be substantially elevated without significantly affecting viability or Wg-regulated cell fates

We next investigated whether Wg signaling was similarly affected by altered APC2 levels—since it is the other key component of the destruction complex and our data revealed that its levels are not substantially different from those of Axin, we suspected it might also be rate-limiting and thus over-expression would inhibit Wg signaling. We used a similar approach to elevate GFP:APC2 levels. Using the MatGAL4 driver, we achieved an ~12-fold increase in APC2 levels (Fig 2D and 2E; S1 Table; hereafter Mat APC2). As we observed with Mat Axin, in Mat APC2 progeny GFP:APC2 levels started high and slowly decreased (Fig 2G). Strikingly, elevating APC2 levels 12-fold had no effect on embryonic viability (94% viable; Fig 3A, S2 Table); in fact, these embryos could develop to adulthood and produce viable offspring. We next examined whether elevating APC2 levels affected Wg-regulated cell fate choices, as assessed by cuticle phenotype. Little or no effect on embryonic patterning was seen (Fig 3B and 3C, S3 Table), and the few denticle belt fusions observed were in hatched larvae. Finally, we examined effects on expression of the Wg target gene *en*. This was also unaffected by overexpression of APC2 (Fig 3D and 3J, S4 Table). Thus, in stark contrast to Axin, embryonic viability, cell fate choice and Wg target gene expression are not sensitive to substantially elevated levels of APC2.

### Elevating levels of APC2 strongly promotes downregulation of the destruction complex in response to physiological levels of Wg signaling

**Fig 5:**
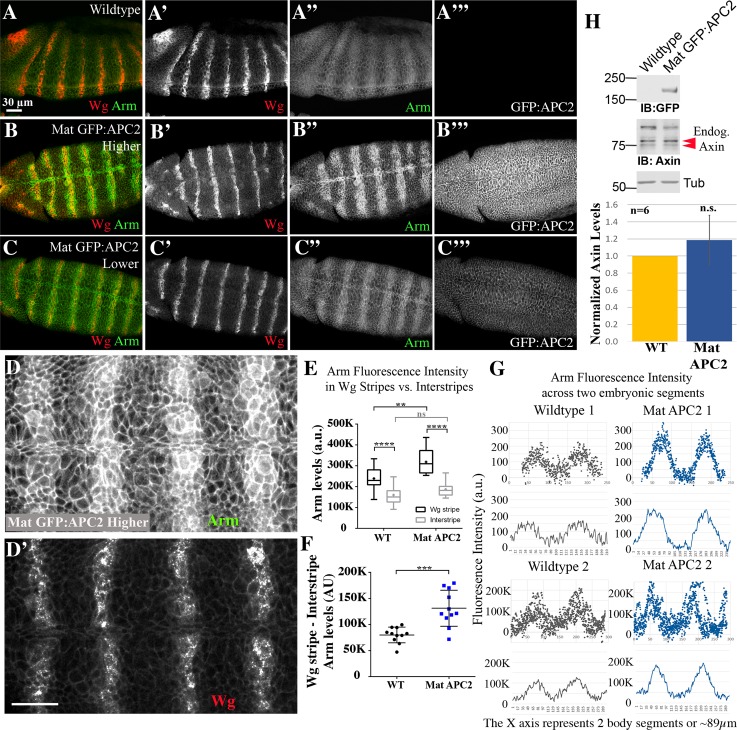
Elevating APC2 levels increases the ability of endogenous Wg signaling to turn down the destruction complex, thus increasing Arm levels in cells receiving Wg. (A-D) Fixed Stage 9 embryos. Anterior to the left. (A-C) Representative images, wildtype (A) or Mat GFP:APC2 embryos with higher (B) or lower (C) levels of GFP:APC2 expression. Elevating APC2 levels increases levels of Arm specifically in cells receiving Wg signal. (D) Close-up, embryo expressing elevated levels of GFP:APC2. The boundary of cells with elevated levels of Arm is quite sharp, and does not expand much farther than the cells adjacent to those expressing Wg. (E) Elevating APC2 levels increases Arm accumulation in Wg stripes but does not affect Arm levels in interstripes. Box and whisker plot (as in Fig 4I and 4K), comparing Arm accumulation levels in Wg-expressing stripes versus Arm in the interstripes in wildtype or Mat GFP:APC2 embryos imaged on the same slide. (F) Difference in Arm accumulation between the Wg stripes and interstripes within individual embryos. Each point = a single embryo. (G) Plots of Arm accumulation pattern over 2 segments. Dot plots = raw data from 3 separate embryos. Line graphs underneath = averages of these data. Elevating APC2 levels exaggerates and sharpens the Arm stripes. (H) Immunoblotting with anti-Axin antibodies reveals that embryos overexpressing APC2 have no change in Axin levels. Statistical analysis: a paired t-test was used to assess the significance between intragroup values in E, and an unpaired t-test was used to determine the significance between intergroup values in E and F. A one-way t-test was used to assess the significance of difference in Axin levels in H. ns, not significant i.e. p≥ 0.05. ** = p<0.01. *** = p<0.001. **** = p<0.0001.

As a final exploration of the effects of elevating APC2 levels, we examined Wg-regulation of Arm stability, using the same assays we employed for analyzing effects of altering Axin levels (S2 Fig). We were surprised to find APC2 overexpression led to a striking change in Arm levels, suggesting reduced activity of the destruction complex. Levels of Arm in Wg-expressing cells and their immediately adjacent neighbors were strongly elevated (Fig 5B–5D vs. 5A), leading to a more defined pattern of stipes in Arm accumulation across each segment. Quantification confirmed that while interstripe Arm levels were unchanged, Arm levels in Wg stripes were significantly higher (Fig 5E and 5F, S5 and S6 Tables). The sharpened stripes and elevated Arm levels in Wg-ON cells were also apparent in our analysis of Arm levels across each segment (Fig 5G). Finally, the same differences were also apparent when we used a membrane mask to examine only cytoplasmic/nuclear Arm or only the membrane pool of Arm (S3A–S3C Fig). These data were quite surprising, as they were the exact opposite of the effects of elevating Axin levels. We examined whether these effects result from reducing Axin levels, a function previously suggested for APC2 [27], but immunoblotting suggested this was not the case (Fig 5H, S2 Table). Instead, these data suggest that when APC2 levels are elevated in a way that accentuates the endogenous APC2:Axin ratio, stabilization of Arm by Wg signaling is enhanced. This could occur by direct effects on the ability of Wg signaling to downregulate the destruction complex, or via the ability of APC2 to bind and sequester Arm [19]—we consider these possibilities more completely in the Discussion. However, this further elevation of Arm levels in cells already receiving Wg signals had little effect on Wnt-target gene expression or cell fate (Fig 3A, 3B and 3D, S2–S4 Tables). Finally, elevating APC2 levels did not alter destruction complex activity in cells not receiving Wg signals, similar to what we observed with Axin (Fig 5E, S3A Fig).

These effects on Arm levels—sharpened and enhanced Arm stripes—were reminiscent of effects previously seen when analyzing APC2 mutants in which the motifs that act as binding sites for Arm (the 15- and 20-amino acid repeats) were reduced in number or eliminated ([44]; S3D Fig). These *APC2* mutants were expressed at endogenous levels in a null *APC2*^*g10*^ background, rather than overexpressed. We analyzed the most extreme of these—*APC2Δ15Δ20R1*,*R3-R5*, which deletes all of the ßcat binding sites, and expressed it in the APC null background = *APC2*^*g10*^ *APC1*^*Q8*^. This allele has a paradoxical phenotype: it strongly reduces APC2 function in Wnt regulation, as assessed by cell fates, but still promotes destruction of Arm in Wg-OFF cells [44]. To determine if the quantitative effects on Arm levels paralleled those we saw after elevating levels of wildtype APC2, we applied our quantitative toolkit to measure Arm levels on Wg-ON and Wg-OFF cells in that mutant. Intriguingly, interstripe Arm levels were unchanged, while Arm levels in Wg stripes were significantly higher (S3E and S3F Fig, S5 and S6 Tables). This phenotype mimics what we observed after elevating levels of wildtype APC2. This may suggest that Wg-ON cells are more sensitive to any perturbation that reduces the function of the destruction complex. We consider the interpretation of this similarity further in the Discussion.

### Simultaneously elevating levels of both APC2 and Axin inhibits Wg signaling more than elevating levels of Axin alone

**Fig 6:**
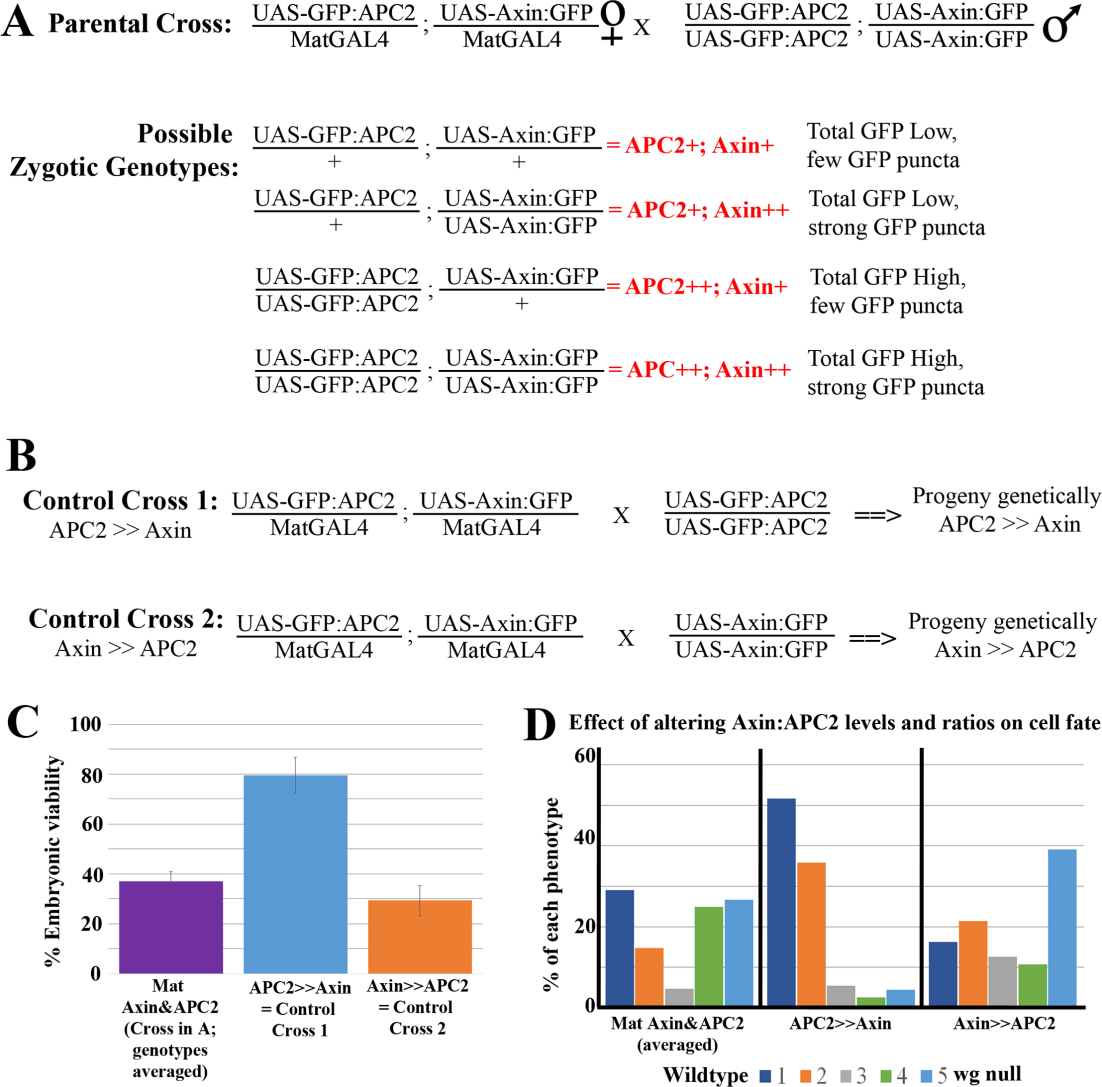
The relative ratios of APC2 to Axin levels determine effects on embryonic viability and Wg-regulated cell fates. (A) Cross used to generate embryos expressing different ratios of APC2 and Axin, with the four categories of progeny, their relative levels of Axin and APC2 overexpression, and the criteria used to identify them. (B) Control crosses used to assess how different ratios of Axin and APC2 overexpression differentially affect embryonic lethality and Wg-regulated cell fate choice. (C) Embryonic viability of different genotypes with differentially altered APC2:Axin ratios. (D) Quantification of the effects of elevating APC2 and Axin on cell fate, as assessed by cuticle pattern. Representative cuticles are in Fig 3C.

These data reveal that elevating Axin levels or elevating APC2 levels had opposite effects on the ability of Wg signaling to regulate destruction complex function. To explore this further, we varied the levels of both proteins simultaneously, and also varied the ratios of their expression levels. We began by expressing both Axin:GFP and GFP:APC2 simultaneously (progeny of GFP:APC2/MatGal4; Axin:GFP/Mat Gal4 females crossed to GFP:APC2; Axin:GFP males; hereafter, Mat APC2&Axin). The progeny of this cross differ in their zygotic genotypes and thus in the relative levels of Axin:GFP and GFP:APC2 (Fig 6A). We first examined the average overexpression levels in embryos including all four zygotic genotypes combined. Immunoblotting revealed that, on average, they accumulate Axin:GFP at levels 4-fold above endogenous Axin (Fig 2H–2J, S1 Table) similar to Mat RFP&Axin (which also contains two UAS transgenes), and accumulate GFP:APC2 at ~20x endogenous levels (S1 Table). However embryonic lethality of embryos overexpressing both Axin:GFP and GFP:APC2 was substantially higher than that of Mat RFP&Axin embryos (63% versus 32% lethal; Fig 6C vs. Fig 3A; S2 Table), despite similar average levels of Axin:GFP accumulation (Fig 2H–2J, S1 Table). In parallel, cell fates were shifted more towards the *wg* null phenotype (Fig 6D, S3 Table) than was seen in Mat RFP&Axin embryos (Fig 3B, S3 Table). Therefore, co-expressing APC2 and Axin inhibits Wg signaling to a greater extent than expressing either Axin or APC2 alone, despite similar average levels of Axin:GFP and GFP:APC2 accumulation (Fig 2H–2J, S1 Table). These data are consistent with the hypothesis that co-expressing Axin and APC2 enhances the resistance of the destruction complex to inactivation by Wg signal.

We suspected that these averages hid differences in outcome among the four different genotypes present among the progeny (Fig 6A), which would express different ratios of APC2 and Axin. To determine which genotypes exhibited elevated embryonic lethality and defects in Wg-regulated cell fates, we set up two additional crosses, in which the relative zygotic expression of Axin and APC2 differed (Fig 6B): 1) APC2>>Axin = average zygotic dose of GFP:APC2 is higher than that of Axin:GFP, and 2) Axin>>APC2 = average zygotic dose of GFP:APC2 is lower than that of Axin:GFP. These two crosses had strikingly different results. APC2>>Axin progeny had only 20% embryonic lethality and Wg-regulated cell fates were only mildly affected (Fig 6C and 6D, S2 and S3 Tables), while Axin>>APC2 progeny had 70% embryonic lethality and had very strong effects on Wg-regulated fates, with 39% having a *wg* null phenotype (Fig 6C and 6D, S2 and S3 Tables). Thus, while APC2 overexpression alone does not affect cell fates, elevating levels of both APC2 and Axin levels inhibits Wg signaling to a greater degree than elevating levels of Axin alone, suggesting both total levels and the relative ratios of Axin and APC2 are important.

### The relative ratio of APC2:Axin levels determines the effectiveness of Arm destruction

**Fig 7:**
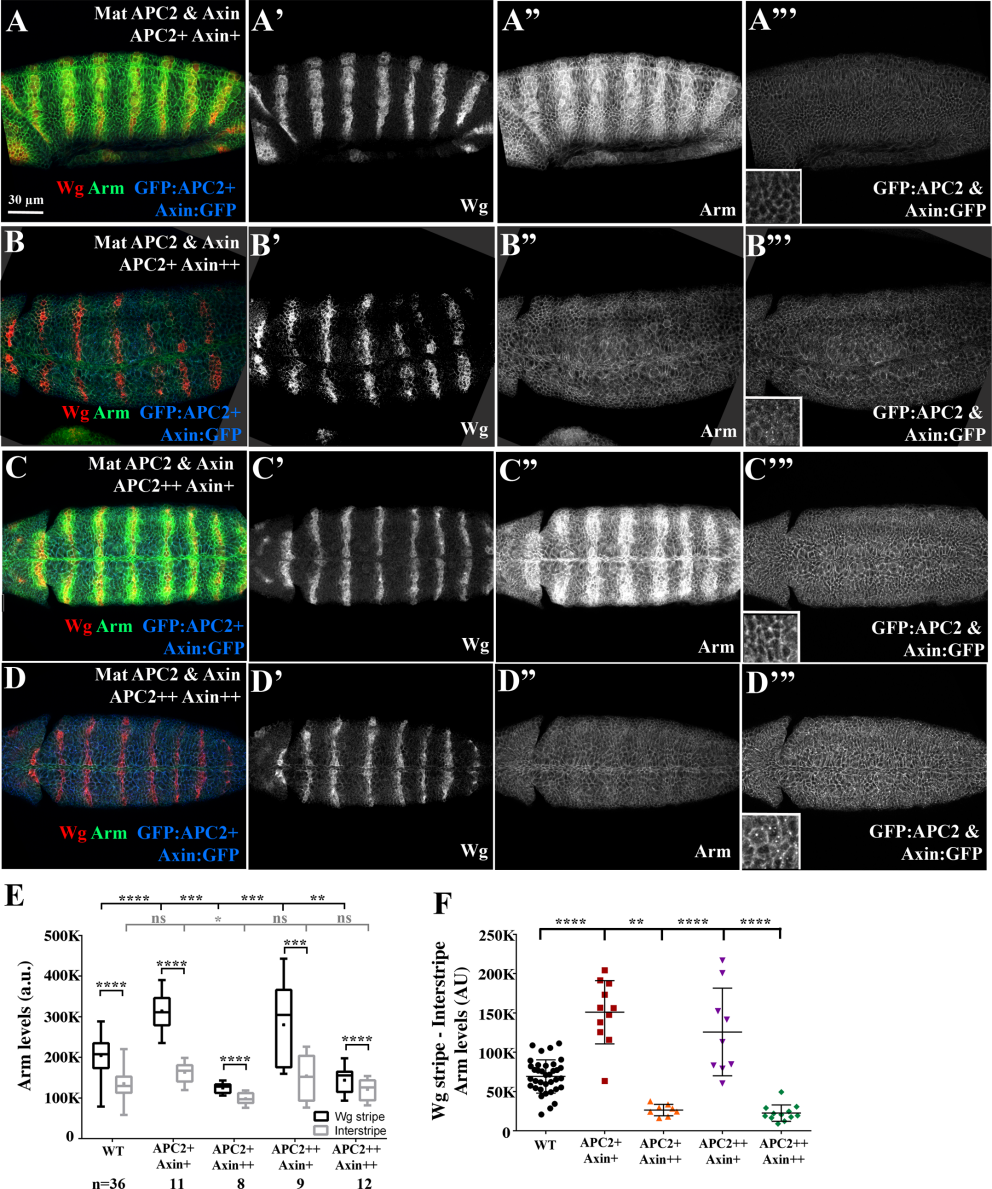
The relative ratios of APC2 to Axin levels determine effects on Arm destruction. (A-D) Fixed stage 9 embryos. Anterior to the left. Representative images of the four different categories of the Mat APC2 & Axin phenotypes from Fig 6. Images were taken under the same microscope settings. Insets are close-ups. See Fig 6A for key to identifying presumptive genotype. (A,C) Both genotypes in which GFP:APC2 elevation exceeds that of Axin:GFP have elevated Arm accumulation in Wg stripes. (B,D) Both genotypes with the highest levels of Axin:GFP have reduced Arm accumulation in Wg stripes. (E) Effects on Arm accumulation in Wg stripes or interstripes in embryos with different ratios of Axin and APC2 accumulation-criteria used to distinguish embryos are in Fig 6. Box and whisker plot (as in Fig 4I and 4K), Arm accumulation in Wg-expressing stripes versus Arm in the interstripes. Wildtype and different presumptive genotypes of Mat APC2&Axin embryos imaged on the same slide. (F) Difference in Arm accumulation between Wg stripes and interstripes within individual embryos. Statistical analysis, a paired t-test was used to determine the significance between intragroup values in E, and an ordinary one-way ANOVA followed by Dunnett's multiple comparisons test was applied between intergroup values in E and F. ns, not significant i.e. p**≥** 0.05. ** = p<0.01. *** = p<0.001. **** = p<0.0001.

These data made strong predictions about how different relative levels of Axin and APC2 would affect Arm destruction. While we could not directly determine genotypes of fixed and stained embryos, we developed a method to infer genotypes from levels and localization of GFP-tagged proteins. Since total protein levels of GFP:APC2 were, on average, higher than those of Axin (Fig 2I, S4A vs. S4B Fig), we first separated embryos into two categories, directly quantifying total GFP expression by immunofluorescence and using low versus high GFP levels as a surrogate for zygotically UAS-GFP:APC2/+ versus zygotically UAS-GFP:APC2/UAS-GFP:APC2 embryos (e.g., Fig 7A’” and 7B’” vs. 7C’” and 7D’”). To further subdivide the embryos, we made use of the assembly of Axin:GFP into cytoplasmic puncta in interstripes [26]. If we could easily visualize cytoplasmic puncta (Fig 7B’” and 7D’” insets), we categorized embryos as zygotically UAS-Axin:GFP/UAS-Axin:GFP rather than zygotically UAS-Axin:GFP/+. This produced four presumptive genotypes with different degrees of overexpression of Axin and APC2 (Fig 6A):

1) APC2+Axin+. Presumptive zygotic genotype = UAS-GFP:APC2/+; UAS-Axin:GFP/+,

2) APC2+Axin++. Presumptive zygotic genotype = UAS-GFP:APC2/ +; UAS-Axin:GFP/UAS-Axin:GFP.

3) APC2++Axin+. Presumptive zygotic genotype = UAS-GFP:APC2/UAS-GFP:APC2; UAS-Axin:GFP/+

4) APC2++ Axin++. Presumptive zygotic genotype = UAS-GFP:APC2/UAS-GFP:APC2; UAS-Axin:GFP/UAS-Axin:GFP.

We then analyzed Arm accumulation in these four embryo categories, using the quantitative tools described above to assess absolute Arm levels in Wg stripes and interstripes relative to wildtype controls. To our surprise, despite the four presumptive genotypes, the embryos divided into two phenotypic categories with regard to Arm accumulation. In embryos of the two genotypes that overexpressed Axin at the highest levels (APC2+Axin++ (Fig 7B); and APC2++Axin++ (Fig 7D)), Arm levels were strongly reduced in the Wg stripes (Fig 7B and 7D: quantified in Fig 7E and 7F; S5 and S6 Tables). Thus, they resembled embryos overexpressing only Axin (Fig 4C, 4D, 4I and 4J). In contrast, the two genotypes that overexpressed APC2 but had lower levels of Axin elevation (APC2+Axin+ (Fig 7A) and APC2++Axin+ (Fig 7C)), Arm levels were strongly elevated in the Wg stripes (Fig 7A and 7C: quantified in Fig 7E and 7F, S5 and S6 Tables). Thus, they resembled embryos overexpressing APC2 alone (Fig 5B and 5C). Combined with the phenotypic data above, these data suggest that the ratio of APC2 to Axin plays a very important role in determining sensitivity of the destruction complex to being inactivated by Wg signaling, such that when Axin is expressed at or over the levels of APC2, the destruction complex is resistant to inactivation, but when levels of APC2 are substantially higher than those of Axin, the destruction complex is more easily inhibited.

### Axin assembles into cytoplasmic multiprotein destruction complexes, and Wnt/Wg signaling leads to their membrane-recruitment and elevates levels of cytoplasmic Axin

One major question still debated in the Wnt field is what happens to the destruction complex after Wnt stimulation. Wnt signaling leads to Axin recruitment to the transmembrane receptor LRP5/6[31]. Work in both cultured human cells and *Drosophila* embryos suggest that both core components of the destruction complex, APC and Axin, can be recruited to the membrane after Wnt stimulation [13,26]. However, three studies of the resulting effects of Wg signaling on Axin levels and localization in the *Drosophila* embryonic epidermis yielded to three distinct conclusions: 1) Wg signaling destabilizes Axin [33], 2) Wg signaling initially stabilizes Axin [28], or 3) Wg signaling leads to Axin membrane recruitment [26].

We thus revisited the question, taking advantage of our ability to express Axin:GFP at defined levels below those at which it significantly inhibits Wg signaling. We first verified that GFP-tagging does not alter physiological roles of Axin: the ability of Axin:GFP to downregulate Arm levels, or its ability to be inactivated in cells that receive Wg. To do so, we expressed Axin:GFP in embryos in which endogenous Axin was knocked down by RNAi (we co-expressed UAS-RFP to account for effects of different copy numbers of UAS–driven transgenes). Axin RNAi led to highly penetrant embryonic lethality (S5A Fig, S2 Table), transformation of cell fates toward Wg-ON fates (as assessed by cuticle analysis; S5 Fig, S3 Table), strong elevation of Arm levels and altered Wg expression (S5C vs S5D Fig). Axin:GFP substantially restored embryonic viability and Wg-regulated cell fate choices (S5A and S5B Fig, S2 and S3 Tables), downregulated Arm and restored normal Wg expression (S5E Fig). Most embryos had a wildtype cuticle, though a small fraction had Wg-signaling inhibited (S5B Fig, S3 Table). Together, these data suggest GFP-tagging does not substantially affect Axin function or its ability to be downregulated by Wg signals.

To further verify that the GFP tag on Axin does not alter its function, we analyzed Axin self-assembly in cultured colorectal cancer cells, where Axin self-assembles into multiprotein “puncta” and recruits APC into these structures [45]. We hypothesize these puncta are larger versions of the normal multiprotein destruction complex [17,19]. Because GFP can dimerize under some conditions, we verified that similar puncta form and recruit APC2 when Axin is tagged with a Flag-epitope rather than with GFP or one of its derivatives (S6A–S6F Fig). We also created a version of Axin tagged with a monomeric mutant of GFP [46], and observed no difference in Axin-self-assembly into puncta or recruitment of APC2 (S6G and S6H Fig). Similar puncta were previously observed in *Drosophila* embryos when Axin:GFP was significantly overexpressed, at levels that inhibit Wg signaling [26]. Membrane-associated endogenous Axin puncta were also seen in imaginal discs, and Axin tagged with the V5 epitope also accumulated in membrane-associated puncta and in the cytoplasm of cells in embryos that received Wg signal [34].

Our system allowed us to directly visualize Axin:GFP localization in embryos expressing it at levels near those of endogenous Axin (Mat RFP&Axin = 4-fold elevated); ~70% of these embryos are viable and >60% have no disruption of Wg-regulated cell fates (Fig 3A and 3B, S2 and S3 Tables). Visualizing Axin:GFP directly avoided issues with antibody accessibility to Axin assembled into large multiprotein complexes versus protein diffuse in the cytoplasm, an issue we observed in cultured colorectal cancer cells ([17]; S6D’ Fig insets).

**Fig 8:**
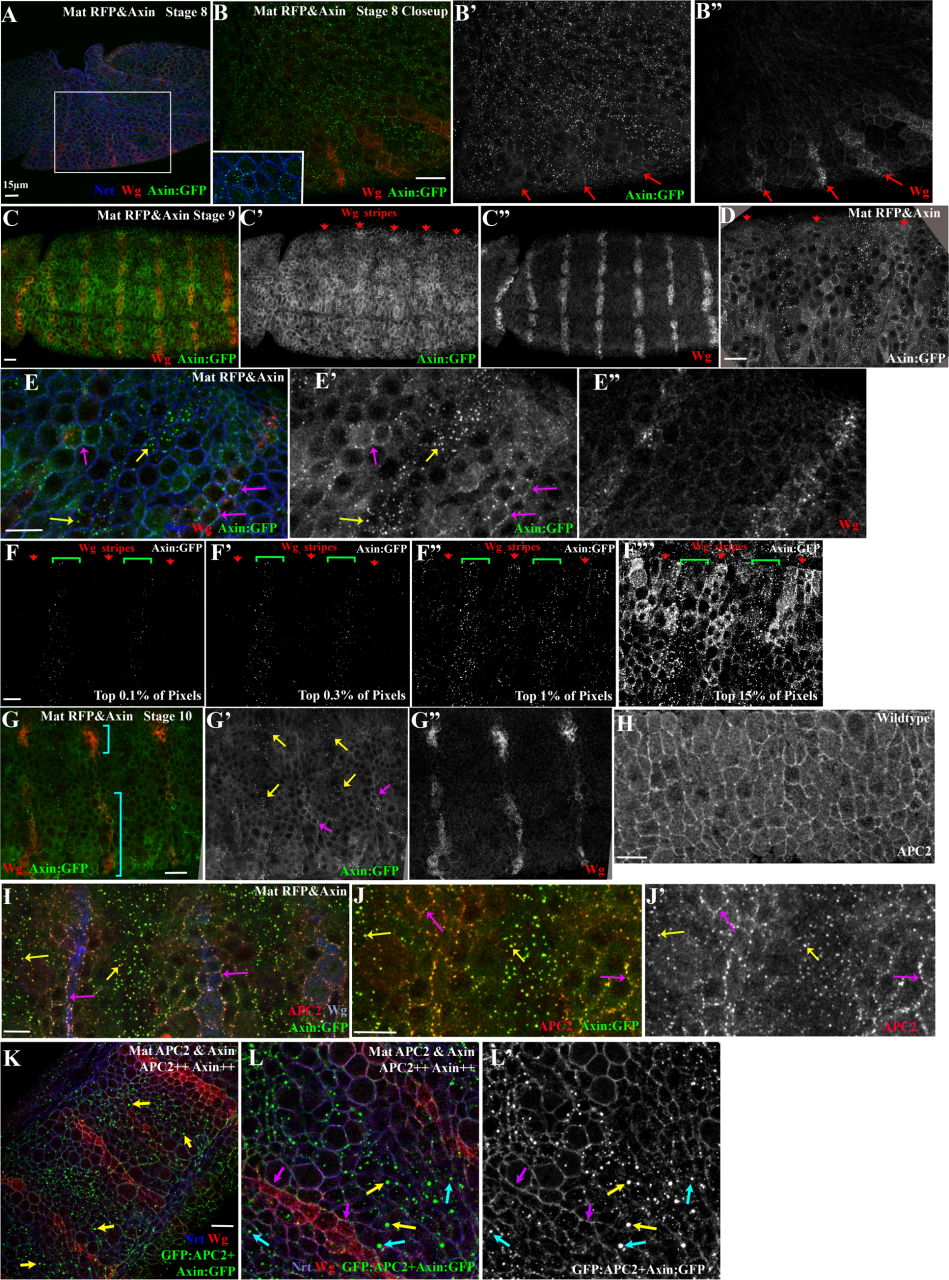
Axin assembles into cytoplasmic multiprotein destruction complexes together with APC2, and Wg signaling leads to their membrane-recruitment and elevates levels of cytoplasmic Axin. (A,B) Fixed stage 8 Mat RFP&Axin embryo. Axin:GFP accumulates in puncta in all cells. Anterior to the left. Inset = close-up of B. Red arrows = initiation of Wg stripes. (C-E) Fixed stage 9 Mat RFP&Axin embryos. Anterior to the left. Red arrows = Wg expressing cells. (D,E) Close-ups of embryo in C showing Axin:GFP localization change in response to Wg. Yellow arrows = cytoplasmic puncta. Magenta arrows = membrane-associated Axin:GFP puncta. (F-F”‘) Image thresholding to determine the relative brightness of different pools of Axin:GFP. (F,F’) The brightest Axin:GFP pixels are in the cytoplasmic puncta in the interstripe cells (brackets). (F”) The next brightest pixels are in membrane–associated puncta in the Wg stripe cells (arrows). (F’”) Diffuse cytoplasmic staining is higher in Wg-stripe cells (arrows) than in interstripes (brackets). (G) Fixed stage 10 embryo. As the Wg stripe separates into medial and lateral domains (brackets), Axin:GFP continues to exhibit differential localization near or distant from Wg-expressing cells. Anterior to the left. Yellow arrows = cytoplasmic puncta. Magenta arrows = membrane-associated Axin:GFP puncta. (H) Localization of endogenous APC2 in a wildtype embryo. (I,J) Expression of Axin:GFP leads to recruitment of endogenous APC2 into both membrane-associated puncta in Wg-ON cells (magenta arrows) and into cytoplasmic puncta in Wg-OFF cells (yellow arrows). (K,L) Mat APC2&Axin. L = closeup. Presumptive APC2++ Axin++ embryo. Simultaneously highly elevating levels of both APC2 and Axin enhances resistance of the destruction complex to be turned off by Wg signaling. Yellow arrow = very bright cytoplasmic puncta. Cyan arrows = bright puncta found near Wg-positive cells. Magenta = membrane-associated puncta in Wg expressing cells. Scale bars = 15μm.

We examined Axin:GFP localization throughout the stages at which Wg signals regulate cell fate. *wg* mRNA expression initiates at the blastoderm stage. As germband extension starts, Wg protein is just beginning to accumulate in stripes (Fig 8A and 8B; [42]). At this stage, most cells had small puncta of Axin:GFP, both membrane-proximal and cytoplasmic, along with a cytoplasmic pool. In some cells near to those initiating Wg expression, Axin:GFP containing puncta were beginning to be enriched at the cortex (Fig 8B arrows). In contrast, at stage 9, when Wg signaling begins to regulate Arm levels and shape cell fate, we observed a prominent difference in Axin:GFP localization in cells receiving or not receiving Wg signal (Fig 8C and 8D). In cells far from the source of Wg, much of the Axin:GFP was assembled into bright cytoplasmic puncta, with relatively low levels in the cytoplasm (Fig 8D and 8E yellow arrows). In contrast, in cells receiving Wg signal, Axin:GFP assembled into less bright membrane-associated puncta, and elevated levels of Axin:GFP were seen in the cytoplasm (Fig 8D and 8E magenta arrows). A similar pattern was observed using GAL4 drivers that led to higher levels of Axin:GFP (*act5c*-GAL4 = Mat/Zyg Axin or MatGAL4 without RFP = Mat Axin). This resembled the pattern previously observed by Cliffe et al. (2003) using a strong GAL4 driver [26]. During stage 10, when the Wg stripes become interrupted, with separate midline and lateral stripes (Fig 8G, brackets), the pattern of Axin:GFP localization became more complex in parallel. Differences in intracellular localization remained between cells near those expressing Wg (Fig 8G, magenta arrows) and those farther away (yellow arrows).

To quantitatively assess levels of Axin:GFP in different subcellular structures, we thresholded our images to different degrees, assessing which structures were brightest and thus likely contained the highest density of Axin:GFP proteins. The results were quite striking. The brightest 0.1% of pixels and most of the brightest 0.3% of pixels, which represent the highest levels of Axin:GFP accumulation, were located in the cytoplasmic puncta in Wg-OFF cells (Fig 8F and 8F’). When we lowered the threshold intensity to visualize the brightest 1% of pixels, the next structures to appear were the membrane-associated puncta in Wg-ON cells (Fig 8F”). It was only when we visualized the brightest 15% of the pixels that the relatively high levels of diffuse cytoplasmic Axin:GFP in the Wg-ON cells were revealed (Fig 8F”‘). This contrasted with the lower cytoplasmic levels of Axin:GFP in Wg-OFF cells.

We next sought to reconcile our observations with recent publications, whose data suggested that the primary effect of Wg signaling was to stabilize Axin in both the cytoplasm and at the membrane [28,34]. These studies used an antibody to an epitope to visualize epitope-tagged Axin. We therefore used a GFP-antibody to visualize Axin:GFP expression (S7A–S7E Fig). Intriguingly, the bright Axin cytoplasmic puncta in the interstripe regions were less apparent (e.g., Fig 8C’ vs. S7A’ or S7C’ Fig)—thus use of an antibody emphasized the stronger cytoplasmic signal in Wg-ON cells, reproducing the earlier observations. This suggested that directly visualizing Axin:GFP provides a more complete picture of the effects of Wg signaling on Axin localization and levels.

Earlier work suggested that when Axin is significantly over-expressed, Axin puncta also contain APC2 [26,47]. We revisited this issue, using our ability to visualize Axin puncta at near endogenous levels (4x-elevated; Mat RFP&Axin) in embryos where Wnt signaling is not substantially inhibited. In wildtype embryos, APC2 is cortically enriched, with a strong cytoplasmic pool (Fig 8H; [35]). Expressing Axin:GFP at 4x endogenous levels significantly altered APC2 localization (Fig 8I and 8J). APC2 was now recruited into both the large cytoplasmic puncta in Wg-OFF cells (Fig 8I and 8J, yellow arrows) and to the smaller, membrane-bound puncta in Wg-ON cells (Fig 8I and 8J, magenta arrows). Intriguingly, recruitment of APC2 into Axin puncta seemed more robust in Wg-ON than in Wg-OFF cells (Fig 8J and 8J’-yellow vs. magenta arrows).

Together, these data suggest that in the absence of Wg signals, Axin self-assembles into large cytoplasmic multiprotein destruction complexes and diffuse cytoplasmic levels of Axin are reduced. Axin recruits APC2 into these puncta and thus they are likely to represent active destruction complexes. In contrast, in cells receiving Wg signal, Axin:APC2 puncta are recruited to the plasma membrane, these puncta diminish in intensity, and the cytoplasmic pool of Axin is correspondingly increased—these changes occur in parallel with and may cause the reduction in destruction complex activity.

### Wg signaling and GSK3 activity are each required for membrane recruitment of Axin puncta

**Fig 9:**
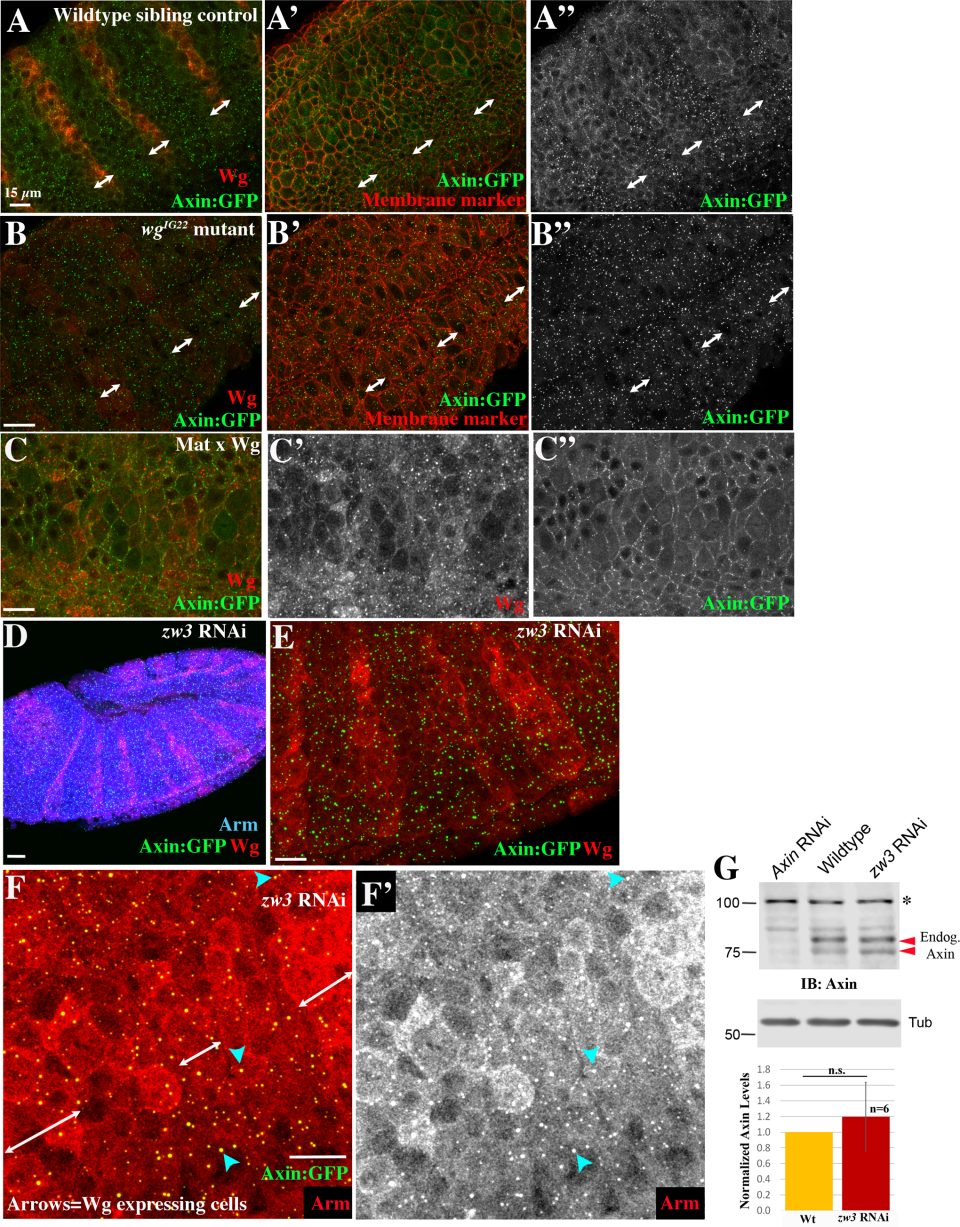
Wg signal and GSK3/Zw3 activity are important for destruction complex membrane recruitment and GSK3/Zw3 regulates release of Arm from the destruction complex. (A,B) Localization of Axin:GFP in stage 9 sibling control embryo (A) and *wg*^*IG22*^ mutant (B). Neurotactin serves as a membrane marker. Both the patterned recruitment of Axin:GFP puncta to the membrane and elevation of cytoplasmic pool of Axin:GFP in Wg-ON cells (double arrows) are lost in *wg*^*IG22*^ mutants. (C) Stage 9 embryo ubiquitously expressing Wg, using the MatGAL4 driver driving both UAS-Wg:HA and UAS-Axin:GFP. Now all cells accumulate Axin:GFP in membrane puncta and also accumulate elevated levels of Axin:GFP in the cytoplasm. (D-F) Stage 9 *zw3* maternal/zygotic RNAi embryos expressing UAS-Axin:GFP, both driven by matGAL4 drivers (= zw3 RNAi x Axin in Methods). (D) Arm levels are highly elevated in all cells. (E,F) Membrane recruitment of Axin:GFP puncta in Wg-ON cells is lost, and Arm accumulates in Axin puncta (F, arrowheads). (G) Immunoblot with anti-Axin antibodies and quantification. Axin levels remain unchanged after *zw3* RNAi (note: UAS:Axin:GFP was not present in this cross = zw3 RNAi in Methods). * = 100 kDa band is non-specific cross-reacting band, as is indicated by the Axin RNAi control. Tubulin was a loading control. A one-way t-test was used to assess the significance of difference in Axin levels. Scale bars = 15μm.

These data suggest that Wg signaling leads to destruction complex recruitment to the plasma membrane, as was observed in cultured cells. To confirm that Wg was required for this response, we visualized Axin:GFP localization in embryos zygotically mutant for the genetically null allele *wg*^*IG22*^ (these mutants produce reduced levels of a non-functional protein, allowing us to identify mutants by reduced Wg accumulation and loss of Arm destruction). Consistent with the hypothesis that reception of Wg triggers membrane recruitment of Axin:GFP puncta, Axin:GFP localized to cytoplasmic puncta in all cells in *wg*^*IG22*^ mutants (Fig 9A vs. 9B), while levels of diffuse cytoplasmic Axin:GFP were relatively low in all cells. These data are consistent with what Cliffe et al. (2003) observed when expressing Axin:GFP at higher levels. We also carried out the converse experiment, using the matGAL4 driver to ubiquitously express UAS-Wg:HA [48], and examined effects on localization of Axin:GFP. Ubiquitous Wg expression led to highly penetrant embryonic lethality and strong expansion of the Wg-regulated naked cuticle fates (S8A, S8B and S8E Fig, S2 and S3 Tables). Ubiquitous Wg expression led all cells to accumulate Axin:GFP in membrane puncta, with elevated levels of Axin:GFP in the cytoplasm (Fig 9C). These data confirm that the alterations of Axin:GFP localization are driven by reception of Wg signal.

The kinase GSK3, encoded in *Drosophila* by the *zw3* gene, plays multiple roles in Wnt signaling [2,31]. In addition to its essential role in regulating Arm/ßcat levels [43,49] by phosphorylating its degron [50,51], GSK3 also phosphorylates the LRP5/Arrow co-receptor, creating Axin binding sites [8]. GSK3 also phosphorylates Axin to regulate its stability and association with ßcat [52,53], and phosphorylates APC on distinct sites to increase its affinity for ßcat [54–56] or to promote ßcat release to the E3 ligase [17]. The most upstream of these roles is in membrane recruitment of the destruction complex via receptor phosphorylation—we thus explored whether reducing GSK3 activity would alter this. We knocked down maternal/zygotic *zw3* by RNAi, and examined Axin:GFP localization. Strikingly, the membrane recruitment of Axin:GFP observed in Wg-ON cells was lost—instead Axin:GFP formed cytoplasmic puncta in all cells (Fig 9D–9F). As expected, Arm levels were strongly elevated (Fig 9D). Moreover, we also observed notable Arm enrichment in the Axin:GFP puncta (Fig 9F). This is intriguing; it is consistent with the role of GSK3 in phosphorylating Arm/ßcat to create an E3 ligase binding site, and with the proposed role of GSK3 in phosphorylating APC2’s R2/B motifs to stimulate transfer of Arm/ßcat from the destruction complex to the E3 ligase [17]. Finally, we saw no significant changes in Axin levels (Fig 9G), suggesting any effects on Axin stability were not substantial.

### Simultaneously elevating Axin and APC2 makes destruction complex puncta more resistant to disassembly by Wg signaling

Our phenotypic data above suggest that simultaneously elevating levels of both Axin and APC2 leads to synergistic inhibition of Wnt signaling. We thus examined how elevating levels of both Axin and APC2 altered destruction complex assembly and localization. GFP:APC2 expressed alone was primarily cortical (Fig 5B and 5C), as observed for endogenous APC2 [35]. In embryos expressing both GFP:APC2 and Axin:GFP at strongly elevated levels (APC2++Axin++ embryos; Fig 8K and 8L), we observed two notable differences from what we observed when each was expressed alone. First, the cytoplasmic puncta in Wg-OFF cells were brighter (Fig 8K and 8L yellow arrows), likely due at least in part to accumulation of two different GFP-tagged proteins into the puncta. Second and more interesting, the region occupied by bright cytoplasmic puncta became much broader, expanding right up to the Wg-expressing cells (Fig 8L, blue arrows), and the region with membrane-associated puncta became narrower, now largely restricted to the single row of Wg-expressing cells (Fig 8L, magenta arrows). Together with the phenotypic data above (Figs 6 and 7), these data suggest that if Axin levels *are limiting relative to those of* APC2 (i.e., APC2>>Axin), the destruction complex is more susceptible to being turned down by Wg signaling. In contrast, if Axin levels *are not limiting relative to those of APC2* (Axin≈APC2), then elevating APC2 levels makes the destruction complex less susceptible to being turned down by Wg signaling. This state correlates with accumulation in large cytoplasmic puncta, consistent with the idea that this occurs by stabilizing destruction complex assembly to the effects of Wg signaling.

### Each destruction complex punctum includes tens to hundreds of APC2 or Axin proteins

Data from both cultured cells and *Drosophila* suggest the ability of Axin and APC to polymerize into a large multimeric complex is critical for targeting βcat for destruction. Polymerization is driven by DIX-domain-mediated head-to-tail Axin polymerization (previously visualized by crystallography and SEM [16]) and by APC’s ability to oligomerize via its N-terminal region and Arm repeats [18]. Overexpressing *Drosophila* Axin in colorectal cancer cells leads to assembly into large “puncta”, which we hypothesize are enlarged versions of the normal destruction complex. APC2 is recruited into these. We used super resolution microscopy to begin to look inside these puncta, revealing that APC2 and Axin form intertwining filaments [17]. To fully understand destruction complex assembly and function, one key parameter is to estimate the number of proteins assembled into active destruction complexes. This has not been possible, either with respect to the large puncta observed after overexpression in colorectal cancer cells, or the presumably smaller complexes produced when Axin and APC2 are expressed at endogenous levels.

**Fig 10:**
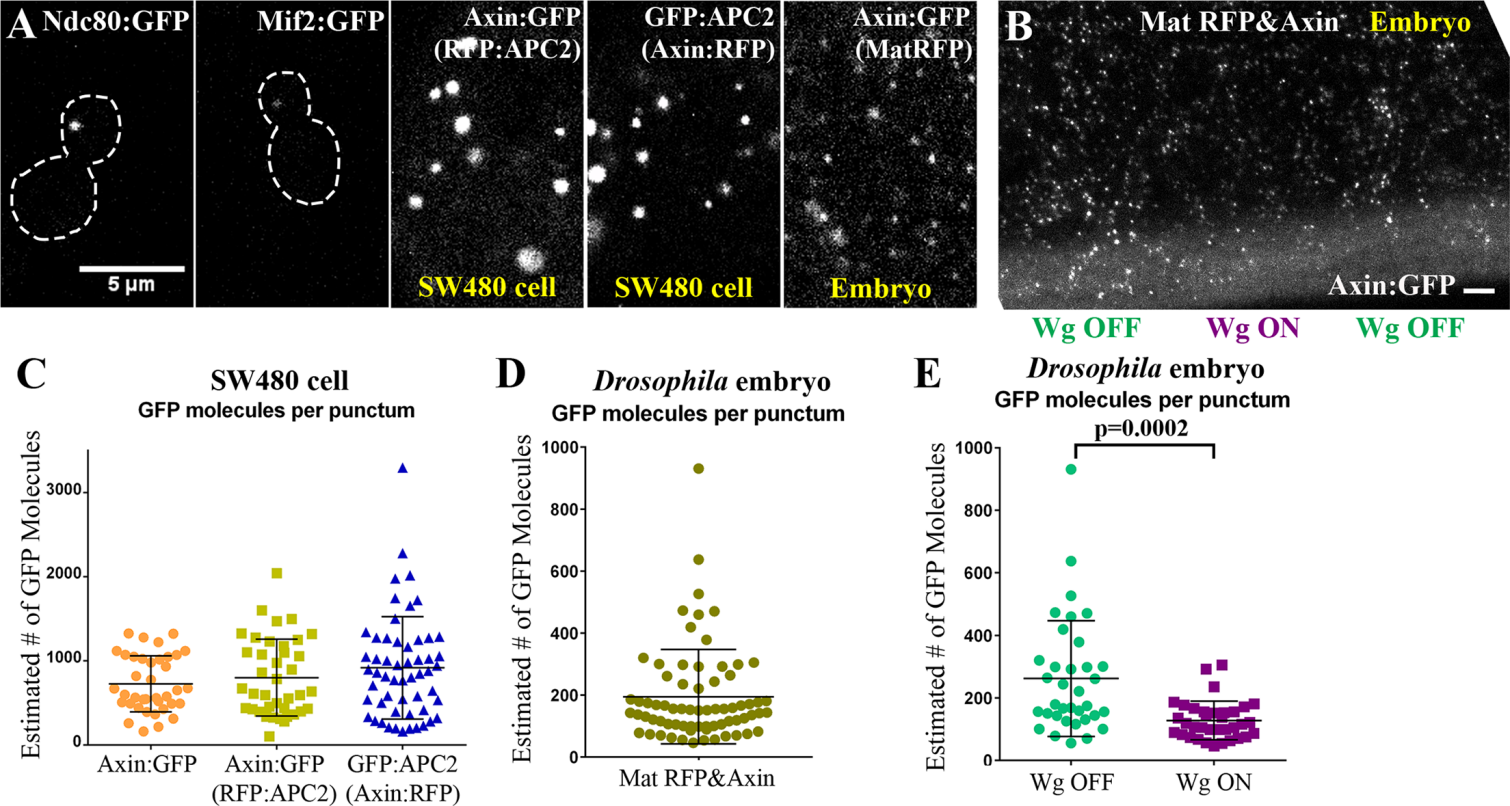
The destruction complex contains thousands of APC2 or Axin molecules after over-expression in SW480 cells, and 10-100s of Axin molecules in vivo in embryos. (A) Representative images of live samples used for fluorescence comparisons to calculate GFP molecule numbers. Each panel is scaled to the same size and brightness. Ndc80:GFP assembles into a structure containing ~306 GFP molecules while Mif2:GFP assembles into a structure containing ~58 GFP molecules. (B) Pattern of Axin:GFP accumulation and localization in a live embryo. Comparison to our fixed samples allowed identification of regions receiving Wg signal (dimmer puncta) or not receiving Wg signal (brighter puncta). (C-E) Estimated number of GFP molecules per punctum. Each dot = an individual punctum analyzed. Means and standard deviation are in S8 Table. (C) GFP Molecule counts from SW480 colorectal cancer cells expressing Axin:GFP alone, Axin:GFP plus RFP:APC2, or GFP:APC2 in addition to Axin:RFP. (D-E) GFP molecule counts *in vivo* from stage 9 embryos expressing RFP and Axin:GFP under the control of MatGAL4 (Mat RFP&Axin). (E) Quantification of puncta GFP molecule counts from D, after being separated into those in presumptive regions receiving or not receiving Wg signals (as in B). Statistical analysis via an unpaired t-test.

To estimate the number of APC2 or Axin molecules within an active destruction complex, we adapted a fluorescence comparison technique developed to quantify numbers of GFP-tagged proteins in multimeric complexes [57,58]. This technique utilized macromolecular structures containing a known number of GFP molecules as standards (e.g., purified eGFP = 2 molecules and a virus-like particle = 120 molecules), and from these developed methods to define the number of proteins in yeast multiprotein complexes where molecule number had not been previously defined. We used 2 yeast strains from this study as standards (Fig 10A): one expressing Ndc80:GFP (calculated to have 306 molecules) and the other expressing Mif2:GFP (calculated to have 58 molecules) [57]. Since we thought it likely that destruction complexes did not have a fixed size, our goal was to get an order of magnitude estimate of the number of proteins in each destruction complex punctum.

We first examined GFP-tagged *Drosophila* Axin over-expressed in SW480 cells. Axin uses its DIX domain to polymerize, forming cytoplasmic puncta in a large range of sizes and brightnesses [17]. We compared living yeast and Axin-expressing SW480 cells in parallel (Fig 10A), using identical imaging conditions (see Methods for details). Puncta size in these cells varies over several orders of magnitude [17], and thus the brightest puncta in each cell exceeded the linear range of our yeast standards and could not be analyzed. We determined brightness of individual puncta and used the two yeast standards to estimate relative brightness and thus relative molecule number. This allowed us to obtain order-of magnitude estimates of the number of Axin molecules per punctum. In the set we analyzed, the number of Axin:GFP molecules per punctum ranged from 163–1327 (mean ~700; Fig 10C; S8 Table). When APC2 is expressed along with Axin in SW480 cells, it is recruited into the Axin puncta [19]. We thus also examined SW480 cells coexpressing both to get order of magnitude comparisons of the number of Axin or APC2 molecules in puncta. In cells co-transfected for Axin:GFP and RFP:APC2, the number of Axin:GFP molecules ranged from 104–2041 (Fig 10C; S8 Table), while in cells transfected with a GFP:APC2 and Axin:RFP, the number of GFP:APC2 molecules per punctum ranged from 162–3297 (Fig 10C; S8 Table), suggesting puncta contain roughly comparable numbers of both proteins. Because the brightest puncta were outside the dynamic range of our camera, and thus were not quantifiable using our yeast standards, these data provide a lower bound for molecule number in the largest puncta. These data suggest that when over-expressed in SW480 cells, APC2 and Axin can assemble into destruction complexes containing at least 100s to 1000s of each protein, and within the complex are likely to be present at the same order of magnitude in molecule number.

While this offered insights into the assembly ability of Axin and APC2, it involved very significant overexpression in an *APC* mutant colorectal cancer cell line. To assess molecule numbers in an active destruction complex in a natural context and at more normal expression levels, we turned to live *Drosophila* embryos from the Mat RFP&Axin line. They express Axin:GFP at 4x-endogenous levels and >60% of these embryos are viable with no or subtle defects in Wg-regulated cell fates (Figs 2A, 2B, 3A and 3B, S1–S3 Tables). We imaged Mat RFP&Axin embryos live, in parallel with yeast expressing each of our two protein number standards (Fig 10A). In embryos even the brightest puncta were within the dynamic range of the camera, and thus could be accurately compared to our yeast standards. Fluorescence comparison revealed that the Axin:GFP puncta range from 46–931 Axin molecules per punctum (at stage 9; average ~200; Fig 10D, S8 Table). As noted above, subcellular localization and apparent brightness of Axin:GFP puncta changed in response to Wg signaling, with the brightest puncta in the cytoplasm of Wg-OFF cells and dimmer, membrane-bound puncta in Wg-ON cells. This difference across the segment was apparent in our live Mat RFP&Axin flies (Fig 10B). We used these criteria to separate the puncta into those in Wg-ON versus Wg-OFF cells. There was a significant difference between the numbers of Axin:GFP molecules in puncta found in Wg-OFF (average ~260 molecules) versus Wg-ON regions (average ~130 molecules; Fig 10E; S8 Table), although the distributions overlapped. These data provide the first insight into the scale of macromolecular assembly in an endogenous destruction complex, suggesting each contains 10s to 100s of Axin molecules. They also support the idea that the number of Axin molecules per destruction complex decreases in response to Wg signaling.

### Dsh accumulates at levels similar to those of APC2 and Axin and localizes to Axin puncta in cells that receive Wg signals

**Fig 11:**
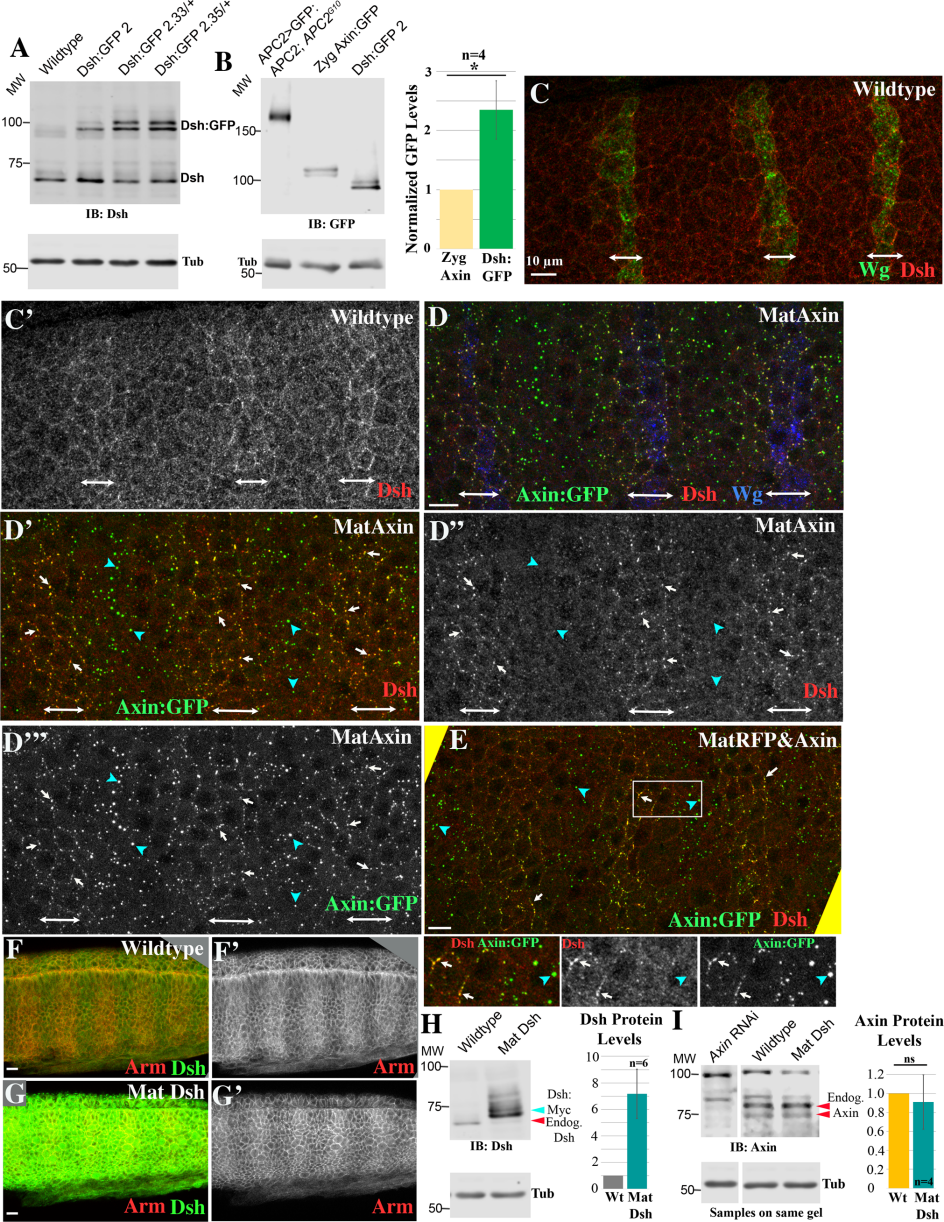
Dsh accumulates at similar levels to Axin and APC2, and co-localizes with Axin puncta in Wg-ON but not Wg-OFF cells. (A) Immunoblot with anti-Dsh antibody, wildtype embryos and three different lines expressing Dsh:GFP driven by its endogenous promotor. Tubulin was the loading control. (B) Left. Immunoblot with anti-GFP antibody, embryos expressing GFP:APC2 driven by its endogenous promotor, Zyg Axin:GFP, or expressing Dsh:GFP driven by its endogenous promotor. Right. Quantification of levels of Dsh:GFP normalized to those of Zyg Axin:GFP. (C-G) Stage 9 embryos, anterior to the left. (C) Dsh localization in a wildtype embryo. There is subtle enrichment of Dsh at the membrane in cells receiving Wg signal (double arrows). (D) In embryos expressing Axin:GFP at 9x endogenous levels (Mat Axin), Dsh co-localizes with membrane associated Axin puncta in Wg-ON cells (white arrows) but not with cytoplasmic Axin puncta in Wg-OFF cells (blue arrowheads). (E) A similar pattern of Dsh recruitment is seen when Axin:GFP is expressed at 4x endogenous levels (MatAxin&RFP). Insets show higher magnification views of region in box. (F,G) Stage 9 wildtype embryo (F) versus embryo overexpressing Dsh (G), using MatGAL4 to drive UAS-Dsh:Myc (Mat Dsh). The elevation of Dsh levels is apparent, but there is little or no effect on Arm regulation. (H) Immunoblot with anti-Dsh antibody. Wildtype embryos versus embryos overexpressing Dsh (Mat Dsh). Tubulin is a loading control. (I) Immunoblot with anti-Axin antibodies and quantification. Axin levels remain unchanged after Dsh overexpression. A one-way t-test was used to assess the significance of difference in Axin levels.

Dsh is a key positive effector of Wnt signaling, acting downstream of the receptors to downregulate the destruction complex. Dsh can co-polymerize with Axin, competing with Axin self-polymerization [10]. Data in vivo suggest Dsh and APC can compete for Axin interaction [47]. This suggested the possibility that these two forms of competition might be part of the mechanism by which Dsh downregulates destruction complex activity. One key factor in evaluating this possibility are the relative levels of the three proteins. Our analysis above revealed that Axin and APC2 accumulate at levels within a few-fold of one another. We adopted a similar strategy to assess the relative levels of Dsh. We obtained a set of Dsh:GFP transgenes driven by the endogenous promotor [59], and used immunoblotting with an anti-Dsh antibody [60] to explore their levels of expression (Fig 11A). We chose the line that accumulated Dsh:GFP at levels closest to endogenous Dsh (line Dsh:GFP 2) and compared accumulation of Dsh:GFP to that of GFP:APC2 and Zyg Axin:GFP (Fig 11B, S1 Table). These data revealed that Dsh accumulates at levels 2.4±0.5 times that of Axin, suggesting that all three proteins are within a few-fold of one another in abundance and thus competition for oligomerization or binding among them are plausible.

We next examined whether endogenous Dsh is recruited into the Axin puncta, either in Wg-ON cells, where it acts to antagonize destruction complex activity, or in Wg-OFF cells where it does not have a known role. We used an antibody to Dsh [60]. In wildtype embryos Dsh was largely cytoplasmic, with weak membrane recruitment in Wg-ON cells (Fig 11C double arrows). However, when we elevated Axin expression (Mat Axin; 9x overexpressed), we saw a striking enhancement of Dsh membrane recruitment in Wg-ON cells. In those cells, Dsh was recruited into membrane-associated puncta that largely co-localized with Axin:GFP (Fig 11D white arrows). In contrast, in Wg-OFF cells Dsh localization remained diffusely cytoplasmic, with little or no enrichment in the cytoplasmic Axin puncta (Fig 11D, blue arrowheads). We saw similar preferential co-localization of Dsh with Axin:GFP in Wg-ON cells when we expressed Axin at near endogenous levels (4x-elevated) such that Wnt signaling is not substantially inhibited (Fig 11E). These data are consistent with co-recruitment of Dsh and the destruction complex to the Wnt receptors in response to Wg signal.

Earlier work revealed that substantially overexpressing Dsh can lead to activation of Wg signaling (e.g. [26,61,62]). Given our new knowledge revealing that relative Dsh and Axin are within a few-fold of one another in abundance, we examined the effect of elevating Dsh levels, using the MatGAL4 driver to express UAS-myc-tagged Dsh ([63]; here after Mat Dsh). This elevated Dsh levels roughly 7-fold (Fig 11F vs 11G and 11H), but had only a modest effect on embryonic viability, reducing it to 83% (S8C Fig, S1 and S2 Tables). Cell fates in most embryos were wildtype, with 29% of the embryos showing mild expansion of naked cuticle (S8D and S8E Fig, S3 Table). At this level of overexpression there was little or no effect on the regulation of Arm levels, as assessed by immunofluorescence (Fig 11F’ vs 11G’), and no apparent effect on levels of Axin, as assessed by immunoblotting (Fig 11I, S1 Table). These data suggest that at endogenous levels of Axin and APC2, Dsh is not strongly rate-limiting for Wnt signaling.

## Discussion

Wnt signaling plays key roles in cell fate choice and stem cell homeostasis in normal development, and mutational activation underlies colorectal and other cancers. The key regulated step in signaling is regulation of the stability of the Wnt effector ßcat by a multiprotein supramolecular machine, the destruction complex. Despite the intense interest in this pathway, the mechanisms by which Wnt signaling regulates destruction complex activity remains a key question in the Wnt field. Our data provide new insights into this in several ways.

### In vivo levels of APC2 and Axin are similar rather than orders of magnitude different

Pioneering work in *Xenopus* egg extracts defined key parameters underlying the biochemical action of the destruction complex, by assembling and measuring destruction complex activity. These studies lacked reagents to directly measure protein levels of all components, and thus used addition of purified Axin to estimate its relative levels. These data suggested Axin is present at levels much lower than the other components of the destruction complex, with an APC:Axin ratio ~5000:1 [22,23]. Many mathematical and other models in the field use these data as an underlying premise, and thus they have been influential in thinking about Wnt signaling.

However, work in cultured mammalian cells cast doubt on the universality of this ratio—in some cell lines APC levels are much more similar to those of Axin (<2-fold higher) while in others Axin was actually present at higher levels than APC [25]. We thus used a well-characterized model where the consequences of Wnt signaling are well known: the *Drosophila* ectoderm during mid-embryogenesis, when cell fate is tightly regulated by Wg signaling. Using both RNAseq and direct comparisons of protein levels, we found that, in contrast to *Xenopus* oocyte extracts, APC and Axin levels are quite similar: our protein data suggest the APC2:Axin ratio is 5:1. In fact, this may overestimate available levels of APC2, the primary APC family member at this time. APC proteins have distinct cytoskeletal roles [64] at times including those just prior to those we examined [65], and thus the pool of APC2 available for Wg signaling may be even lower. While it is possible that the difference in results involve the species used (*Xenopus* vs. *Drosophila*), our data and the mammalian cultured cell data suggest that the difference may be in comparing tissues where Wnt signaling is active, versus those, like *Xenopus* egg extracts, in which Wnt signaling is not yet active. Thus, future mathematical modeling of Wnt signaling should include states in which APC and Axin are present at similar levels.

### In the absence of Wg signaling, Axin assembles into large cytoplasmic multiprotein complexes that each contain tens to hundreds of Axin proteins

Previous work provided conflicting results on *Drosophila* Axin localization in the absence and presence of Wg signaling. In interstripe cells, which receive little or no Wg signal, the destruction complex is in an active state. Cliffe et al. (2003) suggested that Axin and APC2 co-localize in cytoplasmic puncta in these cells [26], while others did not see any notable subcellular localization of Axin in Wg-OFF cells [28,33]. To address this, we examined Axin:GFP localization in embryos expressing Axin below the level that substantially alters embryonic viability or cell fates (Mat RFP&Axin; 4xendogenous). Our data confirm and extend the work of Cliffe et al. [26]. In Wg-OFF interstripe cells, Axin assembled into large cytoplasmic puncta, presumably driven by DIX-domain mediated head-to-tail Axin polymerization, as previously visualized by crystallography and SEM [16]. In these cells, levels of cytoplasmic Axin were relatively low, suggesting that much of the Axin self-assembles into puncta. Earlier data and our work reveal that these puncta also contain APC2 ([26]; Fig 8), and thus APC2’s ability to multimerize may also be relevant [17,18]. Our molecular counting experiments also provided the first assessment of the number of Axin molecules in the multiprotein destruction complex. These data suggest active destruction complexes contain tens to low hundreds of Axin proteins, thus helping explain the critical role of the Axin DIX domain [15,29], which mediates Axin polymerization [16,66]. Our recent work to engineer the minimal Wnt regulatory machine confirmed that both Axin’s DIX domain and APC2’s Arm repeats, implicated in polymerization, are among the domains most critical for destruction complex function [67].

### Wg signaling triggers membrane recruitment of Axin and may destabilize destruction complex assembly

One key and controversial question in the field involves the mechanism(s) by which Wg signaling turns down the destruction complex. Different studies in cultured mammalian cells and *Drosophila* (see Introduction) led to quite different conclusions, ranging from total destruction complex disassembly to inactivation of an intact complex to Axin stabilization. Our new tools allowed us to examine Axin localization directly using a GFP-tagged protein expressed at near endogenous levels, in a tissue where we can examine cells before the onset of Wg signaling, as well as in side-by-side cells experiencing high or low levels of signaling. Our data suggest that in this tissue, Wg signaling leads to membrane recruitment of the destruction complex and are consistent with the idea that it destabilizes assembly, thus increasing the cytoplasmic Axin pool. Before Wg signaling is initiated, Axin:GFP was in cytoplasmic puncta in all cells. However once Wg signaling initiated, Axin:GFP localization differed between cells. In cells not receiving Wg, much of the Axin assembled into large cytoplasmic puncta, leaving relatively low levels diffuse in the cytoplasm. However, in cells receiving Wg signal, the Axin puncta were recruited to the membrane. Our molecular counting and image thresholding experiments suggest these dimmer membrane-proximal puncta contain fewer Axin molecules. Our image thresholding experiments further suggest that in Wg-receiving cells, diffuse cytoplasmic levels of Axin are elevated in Wg-ON relative to Wg-OFF cells.

These data support and extend the earlier work of Cliffe et al. (2003) [26], who expressed GFP-tagged Axin at more elevated levels. Our observation of elevated cytoplasmic levels of Axin in Wg-ON cells is also consistent with earlier work [28]. However, we did not see clear evidence that this results from Axin protein stabilization, as none of our manipulations of Wnt signaling components significantly altered total Axin levels. Our data further suggest that previous use of antibody staining of an epitope-tagged protein rather than direct visualization emphasized the diffuse cytoplasmic pools of Axin in Wg-ON cells while simultaneously de-emphasizing the larger cytoplasmic puncta in Wg-OFF cells, due to differential antibody accessibility. Thus, the stabilization of Axin proposed previously [25] may largely involve a change in protein localization, rather than a change in total Axin levels. This is consistent with the immunoblotting experiments of Cliffe et al (2003), who did not detect altered Axin:GFP levels upon ubiquitous expression of Wg. While Yang et al. 2016 observed an increase in Axin levels by immunoblotting after the onset of Wg signaling in wildtype embryos [28], this simply reflects activation of the zygotic genome.

Our data and earlier data also help define how different components of the Wnt pathway regulate destruction complex localization and assembly. APC2 and Axin co-assemble into large cytoplasmic puncta in Wg-OFF cells. In response to Wg signaling these puncta are recruited to the membrane and reduced in Axin protein number—this recruitment does not occur in *wg* mutants whereas Wg overexpression triggers membrane recruitment in all cells ([26]; Fig 9). Mendoza-Topaz et al. (2011) found that APC2 is critical for assembly of Axin puncta in both Wg-ON and Wg-OFF cells [47]. GSK3/Zw3 is important for membrane-recruitment of Axin puncta, labeling Arm to be recognized by the E3 ligase, and for release of Arm from these puncta (Fig 9). Dsh is specifically recruited into Axin puncta at the membrane of Wg-ON cells (Fig 11), and its overexpression accentuates membrane recruitment of both APC2 and Axin [26]. Integrating our data with this earlier work, we hypothesize that in the presence of Wg signaling, Axin puncta are recruited to the membrane, presumably by binding to the activated Wg-receptor. We further hypothesize that Wg signaling, acting at least in part via Dsh, either destabilizes puncta or inhibits puncta assembly, increasing the relative amount of Axin in the cytoplasmic pool.

### Elevating Axin levels renders the destruction complex less sensitive to inactivation by Wg signaling

Previous work revealed that sufficiently elevating Axin levels could inactivate Wnt signaling either in cultured mammalian cells [68] or in *Drosophila* embryos [26,69], demonstrating that Axin is rate-limiting. More recent work revealed that this only occurred when Axin levels were elevated over a certain level [27,29]. Our knowledge of absolute levels of APC2 and Axin allowed us to vary levels of each individually or together, thus varying both levels and ratios of the two proteins in the *Drosophila* embryo where effects of Wg signaling are well characterized. By assessing the effect on embryonic viability, expression of the target gene *en*, and cell fates choices, we defined the effects of different Axin levels and different timing of Axin accumulation. When Axin:GFP was expressed at ≤4x endogenous Axin, we observed little or no effect on any of these parameters, while at >8x endogenous Axin there was a dramatic increase in embryonic lethality, reduced En expression, and a shift towards a more *wg*-null like phenotype. These data confirm that Axin can be rate-limiting in vivo. Our data also provided insight into the underlying mechanism: increasing Axin ≥4x rendered the destruction complex less sensitive to inactivation by Wg signaling, and thus decreased Arm levels specifically in Wg-ON cells. Further mechanistic insights remain to be determined, but a key parameter may be the levels of “active Dsh” protein, which is activated by Wg signaling and then can heteropolymerize with Axin and compete with APC [16,26,47]. Our data reveal that Dsh levels are in the same order of magnitude as those of Axin, making competition plausible. If down-regulation involves a competition between Axin homo-multimerization and Dsh hetero-multimerization, sufficient elevation of Axin levels may saturate the available Dsh molecules and therefore inhibit its ability to inactivate the destruction complex, thus rendering a subset of destruction complexes immune to downregulation. In contrast, our data suggest Axin is not rate-limiting in Wg-OFF cells—Arm levels there were not further decreased by elevating Axin levels.

### APC2 is not rate-limiting for destruction complex activity but elevating its levels facilitates destruction complex inactivation

We next asked whether APC2, the second core component of the destruction complex, is also rate-limiting for destruction complex activity. Expressing GFP:APC2 at >10x endogenous levels had little to no effect on embryonic lethality, En expression, or Wg-regulated cell fate choices. This might be because Axin is rate-limiting—thus additional APC2 would not trigger assembly of additional destruction complexes once it exceeded the available pool of Axin. Intriguingly, however, we observed an unexpected effect of elevated levels of APC2. In wildtype the normal gradient of Wg creates a gradient of Arm accumulation. In contrast, in embryos with high APC2 expression there is an essentially binary change in Arm accumulation in response to Wg signaling. Wg–expressing cells and their immediately adjacent neighbors accumulate Arm at levels ~1.5x higher than the same cells in wildtype. However, in cells more distant from the Wg-expressing cells, Arm levels are unchanged from wildtype. These data suggest a potential positive role for APC2 in turning the destruction complex down in the presence of Wg signaling. While this paper was under review, another paper was published, suggesting a different role of APC in inhibiting the destruction complex in response to Wg signal, by modulating Axin phosphorylation by GSK3 [70].

### Effects of altering the Axin:APC2 ratio suggest APC2 can play both positive and negative roles in Wnt regulation

These paradoxically opposite effects of elevating levels of Axin or APC2 on the ability of Wg signaling to inactivate the destruction complex were surprising. Our dual over-expression of APC2 and Axin provided potential insight into the underlying mechanisms, emphasizing the importance of the relative ratios of different destruction complex proteins. In embryos with elevated expression of both APC2 and Axin, the destruction complex was even less effectively turned down by Wg signals than after overexpression of Axin alone, and large cytoplasmic Axin/APC2 puncta were found in cells immediately adjacent to those expressing Wg, rather than being confined to cells with lower levels of Wg signaling. As noted above, this may occur in situations where Axin levels exceed those of active Dsh. These data are consistent with the known role of APC proteins in promoting Arm/ßcat destruction, and fit well with our work in cultured mammalian cells, which demonstrated that APC can stabilize Axin multimerization, thus increasing destruction complex size and its effective activity [17].

However, if Axin was limiting, effects of APC2 elevation were quite different. Now elevated levels of APC2 allowed Wg signaling to more effectively turn down the destruction complex, thus elevating Arm levels. It is possible that when APC2 levels exceed those of Axin, it forms incomplete subcomplexes with other destruction complex proteins, thus titrating their levels. Since Axin directly interacts with GSK3 and CK1 while APC2 does not, we do not think the effects of elevating APC2 occurred solely by sequestering those proteins in partially assembled and inactive complexes. One potential explanation is that if APC2 levels exceed those of Axin, it binds and sequesters Arm in binary APC2:Arm complexes, protecting Arm from destruction by the remaining functional destruction complexes. Previous work revealed that the ability of APC2 to retain Arm in the cytoplasm fine-tunes Wg signaling independent of its role in destruction [19,44]. Alternately, these data are consistent with a model in which APC2 has dual positive and negative roles in Wnt regulation, as was previously suggested to occur in the eye and wing imaginal discs [71]. However, the mechanism by which this occurs remains unclear. Our immunolocalization and immunoblotting data do not support the hypothesis that APC2 overexpression promotes Axin turnover, as was previously suggested based on loss-of-function analysis [27,71]. Perhaps the membrane-associated pool of APC2 brings both Axin and the destruction complex in proximity to the Wg receptors and Dsh, allowing more rapid and effective turndown of destruction complex function and assembly.

Our data are also consistent with the hypothesis that relative levels of Axin and active Dsh are key, as we found Axin and Dsh accumulate at quite similar total levels. It would be interesting to examine the effect of varying Dsh levels on destruction complex assembly, localization and function. If Dsh levels are a rate-limiting factor in turning off the destruction complex, then increasing Dsh may balance the effects of elevating Axin. We only saw modest effects of elevating Dsh levels 7-fold, suggesting that elevating Dsh levels alone may not be sufficient, if the ability of the Wg receptor to “activate” the pool of Dsh is limiting. Our data on Dsh localization suggest that its ability to co-localize with Axin requires “activation” by Wg signaling. Moving forward, it would be interesting to count the number of Dsh molecules in the membrane-associated Axin puncta to determine how Dsh molecule numbers within puncta relate to those of Axin or APC2. We also need to explore the ratio of APC2:Axin within the destruction complex *in vivo*, to parallel the work after overexpression in cultured mammalian cells reported here. Wg signaling may alter these ratios and thus regulate destruction complex function. Intriguingly, after Axin over-expression, recruitment of APC2 into Axin puncta seemed more robust in Wg-ON than in Wg-OFF cells (Fig 8J and 8J’-yellow vs. magenta arrows).

One striking aspect of our manipulations was that the effects of elevating levels of APC2 or Axin appeared largely confined to the cells receiving Wg signal. These data are consistent with the idea that normal levels of Axin and APC2 are not rate-limiting in Wg-OFF cells, and thus the alterations in APC2:Axin ratio we created do not alter the efficiency of Arm turnover there. In contrast, the cells receiving Wg signal seem much more sensitive to any manipulation that reduced destruction complex function. These include the elevated APC2:Axin ratio created by elevating APC2 levels, or the reduced ability of APC2*Δ*15*Δ*R1,*Δ*R3-5 to bind and retain Arm in the cytoplasm [44]. One speculative explanation for this is that in those cells, the system is finely balanced, due to competitive interactions between Dsh and Axin, Axin and Axin, and Axin and APC2. In such a scenario, relatively small changes in the levels of any one of these proteins could change the balance of active versus inactive destruction complexes.

### A proposed model of how Wnt signaling regulates destruction complex assembly and function

Our data suggest that the relative levels of different Wnt signaling regulators are a critical determining factor, and that modulating relative levels of different components may make cells more or less sensitive to Wnt signaling. Integrating our data with data from many labs using *Drosophila* and cultured mammalian cells, we propose the following model. During embryogenesis, cells begin with relatively similar levels of the two core scaffolds of the destruction complex (4-5x more APC2 than Axin), and levels of total Dsh protein are in the same range. When Wg signaling is off, Axin self-assembles into cytoplasmic complexes of tens to hundreds of molecules, which we believe represent the functional destruction complex. In this state, much of the Axin in the cell is assembled into puncta, with less free in the cytoplasm. In these cells, we propose that there are 2 pools of APC2, a pool localized to the cortex that mediates APC2’s cytoskeletal functions, and another that is associated with and stabilizes the assembly of the Axin puncta. This would represent a high activity state of the destruction complex, and it would rapidly bind, sequester and turnover all newly synthesized Arm that is not assembled into adherens junctions. In these cells, which lack activation of the Wg receptors, Dsh is not competent to integrate into Axin complexes at levels sufficient to antagonize destruction complex function. In the presence of Wg signaling, LRP5/6 is recruited to the Wg receptor Frizzled, and phosphorylation of LRP5/6 by GSK3 and other kinases recruits Axin and Dsh to the membrane, in the process activating Dsh so it can be incorporated into Axin complexes. We hypothesize that Axin membrane-recruitment involves largely intact destruction complexes. Our observations are consistent with recent data suggesting that the destruction complex is not fully disassembled in response to Wg signaling nor is Arm phosphorylation by the destruction complex completely inhibited [11–13,17]. Instead the ability of the destruction complex to target Arm for destruction is reduced, perhaps in part by blocking the ability to transfer Arm to the E3 ligase. Dsh contains a DIX domain, like Axin, which allows Dsh to hetero-dimerize with Axin. We hypothesize that this Dsh:Axin interaction aids in puncta re-localization and stimulates the decrease in destruction complex size and function. Dsh may actively reduce the size of destruction complexes by competition with Axin:Axin multimerization, or it may compete with APC2 for access to Axin. Other longer-term effects may then reinforce this initial event, including ubiquitination and destruction of Axin or inhibition of GSK3 kinase activity.

## Materials and methods

### Fly stocks, embryonic lethality, and cuticles

All crosses were performed at 25°C. Wildtype was either *y w* or *act5c-Gal4/CyO*. The following stocks were obtained from the Bloomington Stock Center: *act5c-GAL4* (4414), *Maternal alpha tubulin GAL4* (referred to as MatGAL4; a stock carrying both of the GAL4 lines in 7062 and 7063), UAS-Axin:GFP (7225), UAS-Dsh:Myc (9453), UAS-RFP (30556), *wg*^*IG22*^ (5351), UAS-Axin-RNAi (62434), UAS-zw3-RNAi (35364), and UAS-Wg:HA (5918). We also used UAS-GFP:APC2 [19] and an APC2 transgene which expresses APC2 under its endogenous promoter [19]. Dsh:GFP 2, a Dsh:GFP transgene expressed under its endogenous promoter is from [59]. Dsh:GFP 2.33 and Dsh:GFP 2.35, which are derivatives of Dsh:GFP after local gene hopping, were both kind gifts from J. Axelrod (Stanford).

**Cross Abbreviations** (Female x Male):

**GFP:APC2** = *APC2 promoter-GFP*:*APC2; APC2*^*g10*^ *x APC2 promoter-GFP*:*APC2; APC2*^*g10*^

**Zyg Axin** = *UAS-Axin*:*GFP x act5c-GAL4/+*

**Mat RFP&Axin** = *UAS-RFP/MatGAL4; UAS-Axin*:*GFP/MatGAL4 x UAS-RFP; UAS-Axin*:*GFP*

**Mat Axin** = *+/MatGAL4; UAS-Axin*:*GFP /MatGAL4 x UAS-Axin*:*GFP*

**Mat/Zyg Axin** = *act5c-GAL4/+ x UAS-Axin*:*GFP*.

**Mat APC2** = *UAS-GFP*:*APC2/MatGAL4; +/MatGAL4 x UAS-GFP*:*APC2*

**Mat APC2& Axin** = *UAS-GFP*:*APC2/MatGAL4; UAS-Axin*:*GFP/MatGAL4 x UAS-GFP*:*APC2; UAS-Axin*:*GFP*

**APC2 >> Axin** = *UAS-GFP*:*APC2/MatGAL4; UAS-Axin*:*GFP/MatGAL4 x UAS-GFP*:*APC2*

**Axin >> APC2** = *UAS-GFP*:*APC2/MatGAL4; UAS-Axin*:*GFP/MatGAL4 x UAS-Axin*:*GFP*

***Axin* RNAi** = *UAS-Axin-RNAi/MatGAL4; +/MatGAL4 x UAS-Axin-RNAi/+*

**Mat Axin-RNAi x Axin:GFP** = *UAS-Axin-RNAi/MatGAL4; +/MatGAL4 x UAS-Axin*:*GFP*

**Mat Axin-RNAi x RFP** = *UAS-Axin-RNAi/MatGAL4; +/MatGAL4 x UAS-RFP*

**Mat RFP x Axin:GFP** = *UAS-RFP/MatGAL4; +/MatGAL4 x UAS-Axin*:*GFP*

***wg***^***IG22***^ ***mutant** = wg*^*IG22*^*/MatGAL4; +/MatGAL4 x wg^IG22^/+; UAS-Axin:GFP/+*

***zw3* RNAi** = *UAS-zw3-RNAi/MatGAL4; +/MatGAL4 x UAS-zw3-RNAi*

**zw3 RNAi x Axin** = *UAS-zw3-RNAi/MatGAL4; +/MatGAL4 x UAS-Axin*:*GFP*

**Mat x Wg** = *UAS-Wg*:*HA/MatGAL4; +/MatGAL4 x UAS-Wg*:*HA/UAS-Axin*:*GFP*

**Mat Dsh** = *UAS-Dsh*:*Myc/MatGAL4; +/MatGAL4 x UAS-Dsh*:*Myc*

Embryonic lethality assays and cuticle preparations were as in [72]. Inhibition of Wg signaling was assessed by analyzing embryonic and first instar larvae cuticles with the scoring criteria found in Fig 3C and S8 Fig.

### Immunostaining and antibodies

Embryos were prepared as in [73]. Briefly, flies were allowed to lay eggs on apple juice/agar plates with yeast paste for up to 7 hours. Embryos were collected in 0.1% Triton-X in water using a paintbrush, then dechorionated for 5 minutes in 50% bleach. Embryos were fixed for 20 minutes in 1:1 heptane to 9% formaldehyde, with 8mM EGTA added to preserve GFP expression. Embryos were then devitillenized by vortexing in 1:1 heptane to methanol. Embryos were then washed in methanol followed by 0.1% Triton-X in PBS, then incubated in blocking buffer (1:1000 normal goat serum diluted in 0.1% Triton-X in PBS) for 30 minutes. Embryos were incubated in primary overnight at 4°C, washed in 0.1% Triton-X in PBS, then incubated in secondary antibody for 1 hr at room temperature. Embryos were mounted in Aqua polymount (Polyscience). Primary antibodies were: Wingless (Wg, Developmental Studies Hybridoma Bank (DSHB):4D4, 1:1000), Arm (DSHB:N27 A1, 1:75), phospho-tyrosine (pTyr, Millipore:4G10, 1:1000), En (DSHB:4D9, 1:50), GFP (Abcam:ab13970, 1:10,000), Neurotactin (Nrt, DHSB:BP 106, 1:100), APC2 [35], 1:1000), and Dsh ([60]; 1:4000).

### Assessing effects on Engrailed expression

Stage 9 embryos were stained with antibody to Engrailed and imaged on a Zeiss LSM 710 or 880 scanning confocal microscope. Images were processed using FIJI (Fiji Is Just ImageJ) as follows: maximum intensity projections 8μm thick were created and thresholded to highlight cells expressing Engrailed and eliminate background noise. Three lines parallel to the midline were drawn to intersect with bands 2 through 5 of Engrailed expressing cells relative to the head, two on either side of the embryo and one just to the left of the midline. The cells in each Engrailed band which were intersected by each line were included in our measurements. The number of cells per Engrailed stripe was then determined by averaging these three values. Embryos were scored blind. Significance was assessed using a one-way ANOVA test.

### Quantitative analysis of Arm accumulation

#### Graded accumulation of Arm

To quantify effects of our manipulations on the graded accumulation of Arm across the segment, control and experimental embryos were stained in parallel for phosphotyrosine, which marks the adherens junctions, and Arm. Since 70% of Arm protein accumulates at the adherens junction [74], and we wanted to focus on the cytoplasmic and nuclear accumulation of Arm, thus we removed the adherens junction pool of Arm by creating a mask. First, all embryos were rotated to have the anterior on the left and the midline at 180°. Next sum intensity projections were created that went 8μm deep into the embryo. We next used FIJI’s trainable WEKA tool with anti-phosphotyrosine staining to create a membrane mask. This mask was overlaid and subtracted from the Arm image. Next a rectangular region of interest (ROI) was drawn (446 W x 60 H pixels, spanning approximately 3 Wg stripes and 4 cells wide) starting at the first interstripe in the thorax. A profile of the ROI was plotted. ROI profiles were adjusted for embryo length and to bring valleys to zero. See S2 Fig A for a visualization this process.

#### Wg stripes versus interstripes

To calculate the absolute levels of Arm accumulation in cells receiving or not receiving Wg signals, stage 9 embryos were collected and stained as previously described. For each genotype, we added *act5c-*GAL4/Cyo embryos to the same tube as a wildtype control, allowing immunostaining and microscopy imaging on the same slide. Control and experimental embryos were distinguished by the presence or absence of GFP-fluorescence. To calculate the level of Arm accumulation, we choose a specified boxed region (100 pixels wide x 30 pixels high) spanning the width of the Wg-expressing cells, and measured the mean gray value of Arm by FIJI (S2B Fig). Three Wg stripe regions from parasegments 2 to 4 were measured, and the average Arm value minus the background value from a region outside the embryo was defined as the Wg stripe Arm value. In the adjacent interstripe regions we used the same box size to measure and calculate Interstripe Arm values. We also measured the relative difference in Arm accumulation between the Wg Stripes and Interstripes. We also measured the GFP fluorescence of the same boxed regions, allowing us to determine the GFP expression level in different embryos—at times this was used to infer possible genotypes.

To separate and measure the membrane and cytoplasmic pools of Arm, images were rotated so anterior was at the top. A ROI was created (160 W x 20 H pixels, spanning approximately 10–15 cells wide and 2 cells high) which was confined to cells in Wnt OFF or Wnt ON regions. First, a total fluorescence intensity of the ROI was measured and adjusted for background, and then divided by the area of the ROI. Next a membrane mask was created as above and used to measure the intensity and area of the membrane pool of Arm. Next, the membrane mask was used to subtract all of the membrane intensity. Lastly, the remaining intensity in the ROI was measured and recorded as the cytoplasmic/nuclear pool of Arm. To determine the area of the cytoplasmic/nuclear pool of Arm, the area of the membrane was subtracted from the area of the ROI.

### Statistics

Wg-Stripe and Interstripe Arm level values were generally normally distributed, as tested by the D’Agostino-Pearson omnibus normality test as well as the Shapiro-Wilk normality test, and thus parametric tests were employed in statistical analysis. The Paired t-test was used to determine the significance between intragroup values, and an unpaired t-test was used to determine the significance between intergroup values. For multiple comparisons, ordinary one-way ANOVA followed by Dunnett's multiple comparisons test were applied.

### Immunoblotting

4-8hr old embryos were collected in 0.1% Trition-X100, dechorionated in 50% bleach, and then homogenized with a pestle in RIPA buffer (1% NP-40, 0.5% Na deoxycholate, 0.1% SDS, 50mM Tris pH 8, 300 mM NaCl; 1x Halt Protease and Phosphatase Inhibitor (Thermo Scientific)). Protein concentrations were calculated using Protein Assay Dye (BioRad) following the manufacturer’s recommendations. Samples were mixed with SDS-PAGE sample buffer, boiled for 5 minutes and then run on an 8% SDS-PAGE gel and transferred to a nitrocellulose membrane. Westerns were visualized using a CLX Licor machine which allowed blots to be imaged over a 4-log range. Band densitometry was calculated using LICOR Image Studio and significance was assessed using a one-sample *t* test using GraphPad. When band densitometry values differed by more than 5-fold, serial dilutions of samples were used to verify values. Values acquired from these dilutions were reported. Primary Antibodies: anti-GFP (JL-8 Clontech, mouse monoclonal, 1:1000), anti-Axin (a kind gift from Y. Ahmed, guinea pig polyclonal, 1:1000), anti-γ-tubulin (Sigma-Aldrich, mouse monoclonal, 1:2000), anti-APC2 (Rabbit polyclonal, a kind gift of M. Bienz [75], 1:1000), and anti-Dsh ([60], 1:1000). Secondary Antibodies: IRDye680RD anti-Rabbit (Licor, 1:10,000), IRDye680RD anti-Guinea pig (Licor, 1:10,000), and IRDye800CW anti-Mouse (Licor 1:10,000).

### RNA-Seq

mRNA collection and RNAseq analysis are described in [76](GEO accession number GSE38727).

### Cell culture and transfections

SW480 cells were cultured at 37° C at normal atmospheric levels of CO_2_ in L15-media (Cellgro) supplemented with 10% FBS and 1x penicillin–streptomycin. *Drosophila* APC2 or Axin protein constructs were transfected into SW480s using Lipofectamine 2000 (Invitrogen) as recommended by the manufacturer. Cells were imaged 24 hours later. Full length *Drosophila* APC2 and Axin were cloned with either a GFP, RFP, or Flag tag as in [17].

To verify that Axin:GFP polymerization is not simply a result of di- or oligomerization of the GFP protein, we created a monomeric GFP (mGFP) by changing Alanine 206 to Leucine [46]. We created the A206K amino acid change in our base plasmid (pCMV-Axin:GFP; [17]) using QuikChange (Agilent Technologies) following manufacturer’s recommendations. The full plasmid was sequenced to verify the amino acid change in GFP and to ensure no other detrimental mutations were induced in the plasmid.

### Yeast fluorescence comparison

Yeast Fluorescence comparison analysis was performed as described in [57,58]. Briefly, yeast were grown at 24°C in YPD media until they reached an OD between 400–600. Yeast cells were then pelleted and resuspended in YC complete media for live imaging. To adjust for possible background caused by media, both SW480 cells and embryos were also suspended in YC complete medium for live imaging. Images were taken using the same settings on the same day for each experiment. Each slide was imaged for no longer than 20 minutes at room temperature (~25°C). For analysis, a 15 x 15pixel ROI was created around each punctum, then a 21 x 21 pixel ROI was made around the smaller ROI for background subtraction. Puncta distance from the coverslip and depth of field (number of Z slices containing a single punctum) were both taken into account when calculating the molecule numbers (as in [58]). To verify molecular counting, each punctum was compared to 2 different yeast strains: Ndc80:GFP (~306 molecules of GFP) and Mif2:GFP (~58 molecules of GFP; both kind gifts from K. Bloom [57]). Data sets were only used when molecular numbers were consistent (+/- 15 molecules) when calculated with both the Ndc80 and Mif2 standards. Due to the dynamic range of the camera, we were limited in the brightness of puncta that we were able to image in SW480 cells. Therefore, the brightest (presumed largest) puncta were omitted from analysis, suggesting that our molecular counts in SW480 cells are an underestimate, as noted in the Results. However, all puncta in embryos were within the camera’s dynamic range. Images were taken on a Zeiss LSM 710 confocal microscope using a Licor LSM-T-PMT camera, with a 488nm diode for stable illumination on a 100x/1.4 NA objective lens. Images were analyzed using FIJI. For a more detailed description of how to calculate molecular number see [58].

## Supporting information

S1 Fig Crosses used to achieve different level and timing of Axin elevation. Crosses and expected progeny are diagrammed, along with features of expression characteristic of the GAL4 driver used.
(TIF)

S2 Fig Assessing gradation of Arm levels across the segment and absolute levels of Arm in Wg stripes and interstripes. (A-A”) Representative example illustrating assessment of graded cytoplasmic and nuclear Arm levels across 2–3 embryonic segments (A) Original image of Arm (A’) Plasma membrane mask created using pTyr staining of the same embryo in A. (A”) Resulting Arm image after subtracting A’ from A. The green box illustrates a region of interest (ROI) selected. A profile of the ROI was plotted along the anterior-posterior axis. ROI profiles were adjusted for embryo length and to bring valleys to zero. (B-B”) Representative example illustrating calculations of absolute levels of Arm in Wg-expressing cells (Wg-stripes) and Wg-OFF cells (interstripes). Yellow boxes indicate regions sampled for Wg stripes. White boxes represent the regions sampled for the interstripe regions. The blue box represents the region sampled for background. In all cases, wildtype and mutant embryos were imaged together using constant imaging conditions.
(TIF)

S3 Fig The opposite effects of Axin versus APC2 overexpression on Arm levels in Wg-ON cells are observed in both the cytoplasmic and membrane-associated pools. We separately assessed how elevating levels of Axin or APC2 affected total Arm levels (A), levels of Arm in the cytoplasmic/nuclear pool (B), or levels of Arm in the junctional (membrane) pool (C), by using a membrane marker to create an image mask (see Methods). Elevating Axin levels 9-fold (Mat Axin) reduced Arm levels in each of these pools in Wg-ON cells, without affecting levels of Arm in any of the pools in Wg-OFF cells, relative to wildtype. Conversely, elevating APC2 levels 11-fold (MatAPC2) increased Arm levels in each of these pools in Wg-ON cells, without affecting levels of Arm in any of the pools in Wg-OFF cells, relative to wildtype. (D) Embryo expressing a mutant APC2 protein deleting all of the ßcat binding sites (APC2Δ15Δ20R1,R3-R5 (expressed in the APC null background = *APC2*^*g10*^ *APC1*^*Q8*^; referred to here as APCΔßcat). Arm accumulation is enhanced in Wg-ON cells without affecting accumulation in Wg-OFF cells. (E) Quantification of Arm levels. (F) Difference in Arm levels between Wg stripes and interstripes. Statistical analysis: a paired t-test was used to determine the significance between intragroup values in E, and an unpaired t-test was used to determine the significance between intergroup values in E and F. ns, not significant i.e. p≥ 0.05. ** = p<0.01. *** = p<0.001. **** = p<0.0001.
(TIF)

S4 Fig Illustration of how embryos were sorted as to inferred genotype. (A,B) Fixed stage 9 embryos imaged under the same microscope conditions, illustrating that when expressed using the MatGAl4 drivers, GFP:APC2 is substantially brighter under our conditions than Axin:GFP, allowing us define GFP:APC2 genotypes by overall GFP fluorescence. (A) GFP brightness of Mat Axin embryo. (B) Example of a MatAPC2 & Axin embryo we scored in the GFP high category. This embryo would also have been scored as Axin high because of the bright puncta.
(TIF)

S5 Fig Axin:GFP can largely restore normal Wnt signaling after *Axin* RNAi. We compared the effects of Axin RNAi with or without expressing Axin:GFP. Crosses: matGAL4/+; matGAL4/Axin shRNA females to either UAS-Axin:GFP males or UAS-RFP males as a control. We also crossed matGAL4/UAS:RFP; matGAL4/+ females to UAS-Axin:GFP males to control for effects of Axin:GFP expression. (A) Assessment of embryonic viability. Axin RNAi leads to highly penetrant embryonic lethality which is largely rescued by expression of Axin:GFP. (B) Assessment of effect on Wg regulated cell fates via cuticle analysis. Categories are illustrated in Fig 3 (reduced Wg signaling) or S8E Fig (increased Wg signaling). Axin-RNAi expands the Wg-promoted naked cuticle fates. This is largely rescued to wildtype by expression of Axin:GFP, though a few embryos lose naked cuticle, as is seen in the control expressing only Axin:GFP. (C-F) Stage 9 embryos, visualize Wg, Arm and Axin:GFP. (C) Wildtype. (D) Axin-RNAi. Note elevated Arm levels and expansion of Wg stripes. (E) Axin-RNAi combined with expression of Axin:GFP. The normal segmental stripes of Arm and the single-cell wide stripes of Wg expression are restored. (F) Expression of Axin:GFP. At this level of expression most embryos have near normal Arm stripes.
(TIF)

S6 Fig Flag-tagged Axin assembles into puncta indistinguishable from those assembled by Axin:GFP. Constructs expressing the indicated proteins were transfected into SW480 cells and visualized either using the fluorescent tag or using an anti-Flag epitope antibody. (A,C,E,G) Axin:RFP (A), Flag:Axin (C), Axin:GFP (E), and Axin:monomeric GFP (mGFP) (G) all assemble into numerous puncta-no differences were seen in this regard (B) RFP:APC2 is diffusely cytoplasmic. (D,F,H) Flag:Axin (D), Axin:GFP (F) and Axin:mGFP (H) can all recruit RFP:APC2 into puncta. Insets = closeups of puncta, illustrating co-localization.
(TIF)

S7 Fig When Axin is localized using an antibody to the GFP epitope-tag, it emphasizes the elevation in cytoplasmic Axin in Wg-ON cells and de-emphasizes Axin puncta in Wg-OFF cells. (A-D). Stage 9 embryos, anterior to the left. (E) Late stage 9/stage 10 embryo. All are expressing Axin:GFP using the matGAL4 driver (Mat Axin) and all stained with antibodies to GFP and Wg, along with Neurotactin (Nrt) to visualize the plasma membrane. B and D are close-ups of A and C, respectively. (A,C) Antibody staining clearly reveals elevated cytoplasmic Axin:GFP in cells receiving Wg signal (arrows). (B) In optimally stained embryos, close-ups also reveal both cytoplasmic puncta in Wg-OFF cells (yellow arrows) and membrane-associated puncta in Wg-ON cells (magenta arrows). (D) In many embryos cytoplasmic puncta in Wg-OFF cells are either not visible or less apparent (yellow arrows), while membrane-associated puncta in Wg-ON cells remain visible (magenta arrows) and elevation of cytoplasmic Axin in Wg-ON cells becomes prominent. (E). By late stage 9-early stage 10, we can visualize the decrease in cytoplasmic Axin in cells expressing Wg (arrows), as was previously reported [27].
(TIF)

S8 Fig Ubiquitous expression of Wg increases embryonic lethality and induces a loss of denticle belts, whereas Dsh overexpression has little effect on viability and cuticle phenotype. (A) Ubiquitous expression of UAS-Wg:HA using the MatGAL4 driver (Mat Wg) reduces embryonic viability to 1.2%. (B) Ubiquitous Wg expression promotes Wg-regulated cell fates and thus reduces the number of denticle belts formed. (C) Overexpressing UAS-Dsh:Myc using the MatGAL4 driver (Mat Dsh) slightly reduces embryonic viability (83.3 +/- 7.3%). (D) >60% of embryos and larva have a wildtype cuticle phenotype while 29% have a partial loss of denticle belts. (E) Representative images of the phenotypic categories used in B and D. Category 0 = wildtype; Category -1 = At most one denticle belt, or part of 2 belts are absent (arrow); Category -2 = more than half of total denticle belts are present (arrows represent lost denticle belts); Category -3 = less than half of total denticle belts remain, strong head defects or head hole (arrow); Category -4 = A small patch of denticle to none, head hole (arrow).
(TIF)

S1 Table Normalized densitometry values. Quantification of protein levels of endogenous and GFP-tagged proteins via immunoblotting followed by band quantification using the CLX Licor, which allows samples to be quantified in a 4-log range. When differences were ≥ 5-fold, dilutions were used to verify levels. Crosses and their abbreviation are labelled. Means, standard deviation, and number of blots quantified are indicated. Significance was calculated using a one-sample *t-test*.
(XLSX)

S2 Table Embryonic viability. Quantification of embryonic viability after altering levels of GFP:APC2 and/or Axin:GFP, Wg:HA, Dsh:Myc, or Zw3. Crosses, embryonic viability, standard deviation and numbers of embryos assayed are indicated.
(XLSX)

S3 Table Embryonic and first instar larva cuticle phenotype. (A)Effects of GFP:APC2 and/or Axin:GFP, Wg:HA, Dsh:Myc, or Zw3 manipulations on embryonic and first instar larva cuticle phenotypes. Fig 3C show the cuticle categories. (B) Effects of Axin shRNA, overexpression of Wg or Dsh on embryonic and first instar cuticle phenotypes. Fig 3C and S8 Fig E show the cuticle categories. n = number of embryos/larva scored.
(XLSX)

S4 Table Rows of En-expressing cells per segment. Quantification of the number of rows of En expressing cells per segment, in embryos in which APC2 or Axin levels are decreased or elevated. n = number of embryos scored. Significance was determined using one-way ANOVA with GraphPad.
(XLSX)

S5 Table Effects on Arm levels of elevating Axin and/or APC2 levels. Arm accumulation levels in Wg-expressing stripes versus Arm levels in the interstripes for the indicated genotypes as in S2 Fig B. These are the raw data used to create the box-and-whisker plots in the Figures. n = number of embryos examined.
(XLSX)

S6 Table Quantification of the difference in Arm levels in Wg-stripe versus interstripe-cells. Quantification of difference in Arm accumulation between the Wg stripes and interstripes within individual embryos. These are the raw data behind the scatter plots in the Figures. n = number of embryos examined.
(XLSX)

S7 Table Quantification of the different pools of Arm levels in Wg-stripe versus interstripe-cells. Quantification of difference in Arm accumulation between the Wg stripes and interstripes within individual embryos. These are the raw data behind the scatter plots in the S3 Fig. n = number of segments analyzed.
(XLSX)

S8 Table Fluorescence comparison values. Detailed results from calculating the number of GFP-tagged Axin or APC2 proteins in Axin puncta in live human SW480 colon cancer cells or in *Drosophila* embryo. These data form the basis of Fig 9. n = number of puncta examined.
(XLSX)
