# Supplementary figures and images for: Supramolecular assembly of the beta-catenin destruction complex and the effect of Wnt signaling on its localization, molecular size, and activity in vivo
Source: PLoS Genet. 2018 Apr 11;14(4):e1007339. doi: 10.1371/journal.pgen.1007339 (PMC5912785; doi:10.1371/journal.pgen.1007339)

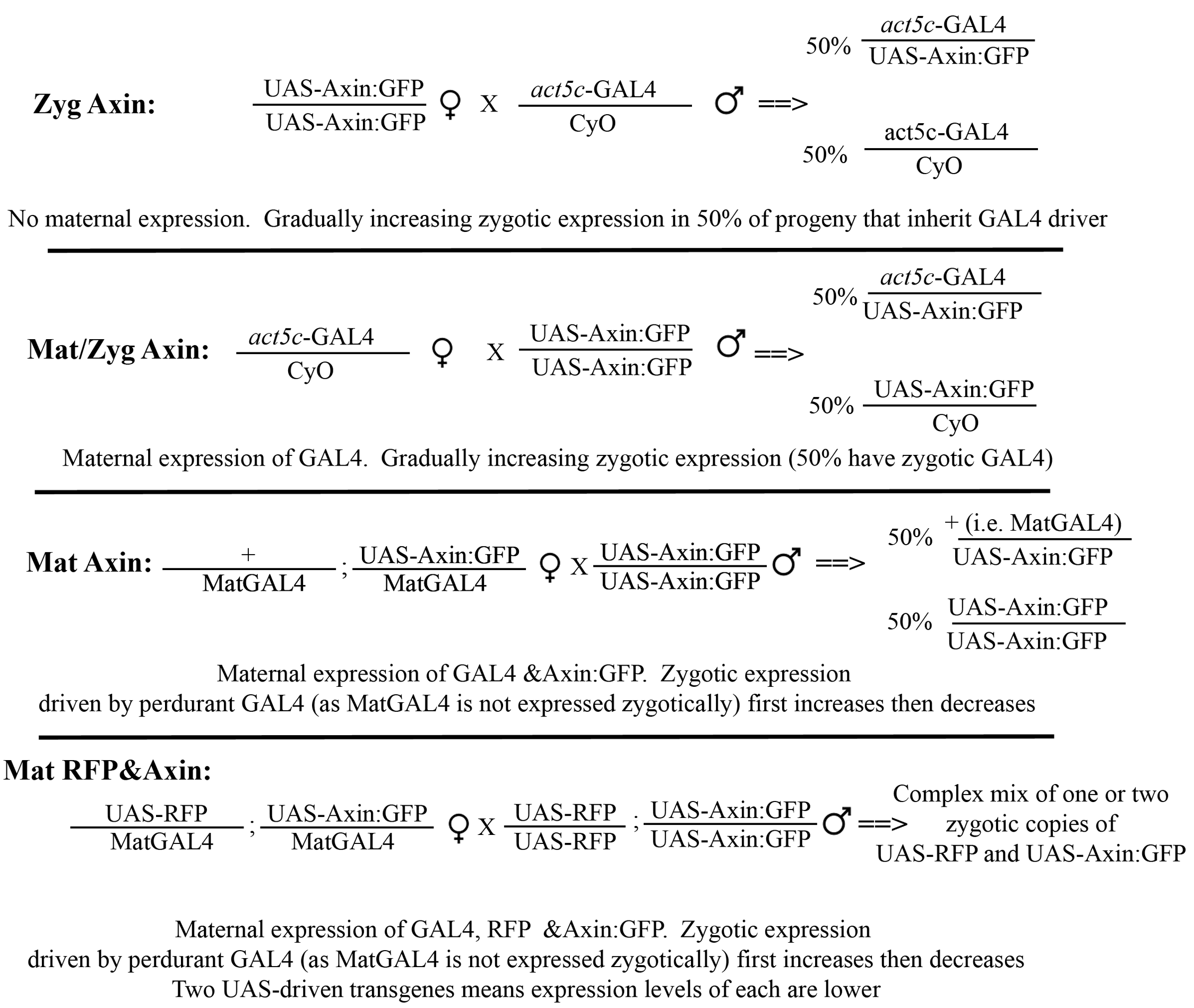

Supplement: S1 Fig — Crosses and expected progeny are diagrammed, along with features of expression characteristic of the GAL4 driver used. (TIF) [file pgen.1007339.s001.tif]

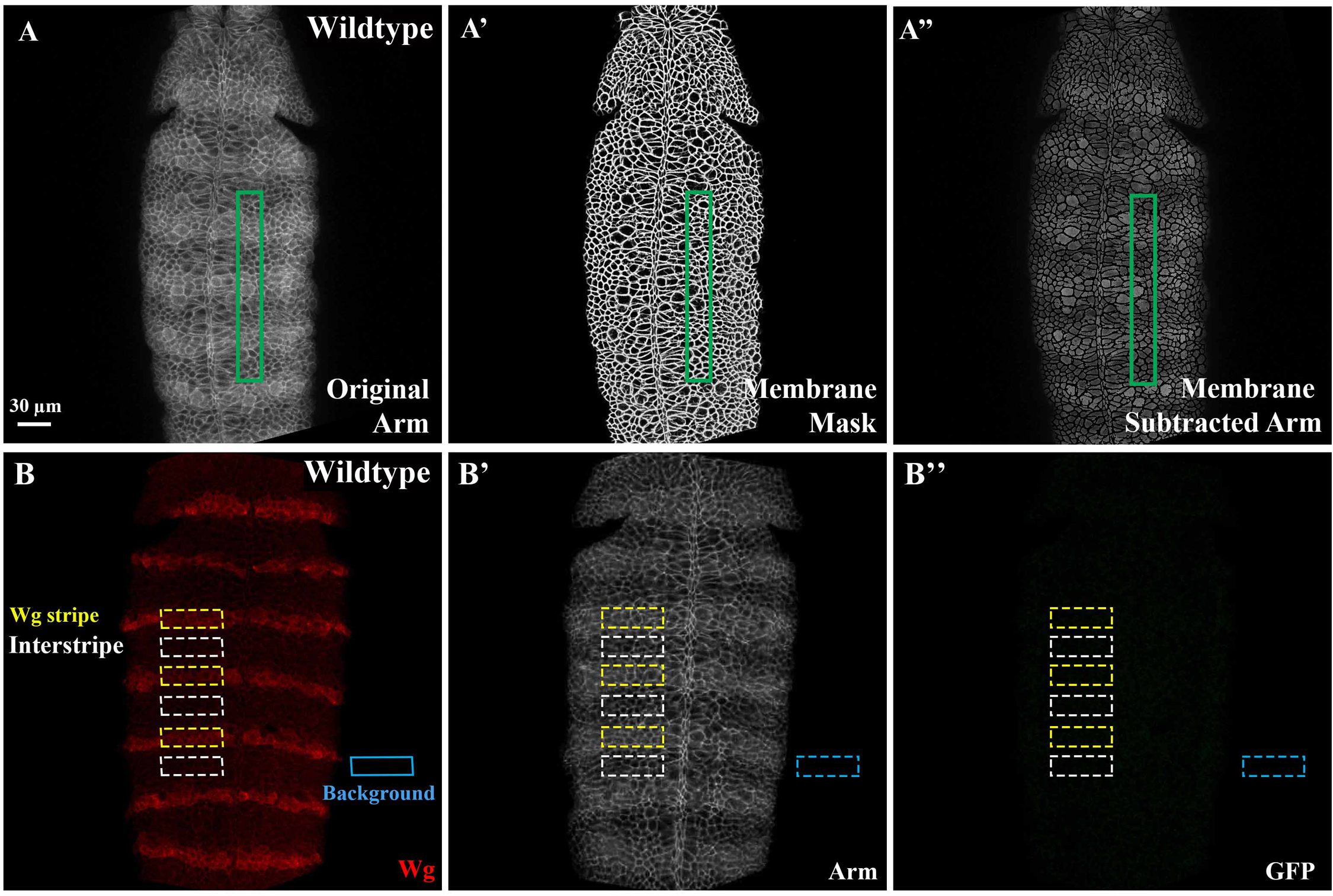

Supplement: S2 Fig — (A-A”) Representative example illustrating assessment of graded cytoplasmic and nuclear Arm levels across 2–3 embryonic segments (A) Original image of Arm (A’) Plasma membrane mask created using pTyr staining of the same embryo in A. (A”) Resulting Arm image after subtracting A’ from A. The green box illustrates a region of interest (ROI) selected. A profile of the ROI was plotted along the anterior-posterior axis. ROI profiles were adjusted for embryo length and to bring valleys to zero. (B-B”) Representative example illustrating calculations of absolute levels of Arm in Wg-expressing cells (Wg-stripes) and Wg-OFF cells (interstripes). Yellow boxes indicate regions sampled for Wg stripes. White boxes represent the regions sampled for the interstripe regions. The blue box represents the region sampled for background. In all cases, wildtype and mutant embryos were imaged together using constant imaging conditions. (TIF) [file pgen.1007339.s002.tif]

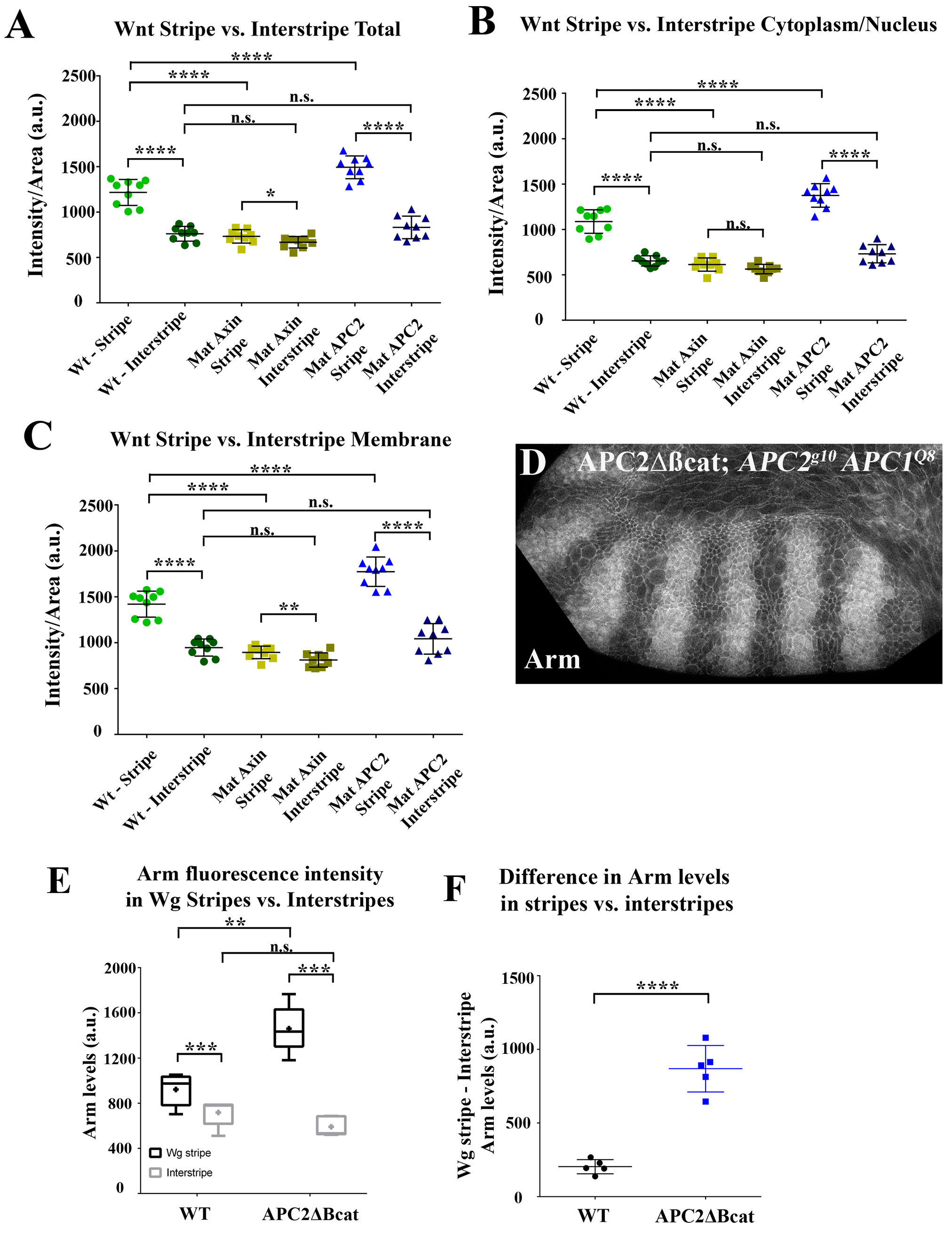

Supplement: S3 Fig — We separately assessed how elevating levels of Axin or APC2 affected total Arm levels (A), levels of Arm in the cytoplasmic/nuclear pool (B), or levels of Arm in the junctional (membrane) pool (C), by using a membrane marker to create an image mask (see Methods). Elevating Axin levels 9-fold (Mat Axin) reduced Arm levels in each of these pools in Wg-ON cells, without affecting levels of Arm in any of the pools in Wg-OFF cells, relative to wildtype. Conversely, elevating APC2 levels 11-fold (MatAPC2) increased Arm levels in each of these pools in Wg-ON cells, without affecting levels of Arm in any of the pools in Wg-OFF cells, relative to wildtype. (D) Embryo expressing a mutant APC2 protein deleting all of the ßcat binding sites (APC2Δ15Δ20R1,R3-R5 (expressed in the APC null background = APC2g10 APC1Q8; referred to here as APCΔßcat). Arm accumulation is enhanced in Wg-ON cells without affecting accumulation in Wg-OFF cells. (E) Quantification of Arm levels. (F) Difference in Arm levels between Wg stripes and interstripes. Statistical analysis: a paired t-test was used to determine the significance between intragroup values in E, and an unpaired t-test was used to determine the significance between intergroup values in E and F. ns, not significant i.e. p≥ 0.05. ** = p<0.01. *** = p<0.001. **** = p<0.0001. (TIF) [file pgen.1007339.s003.tif]

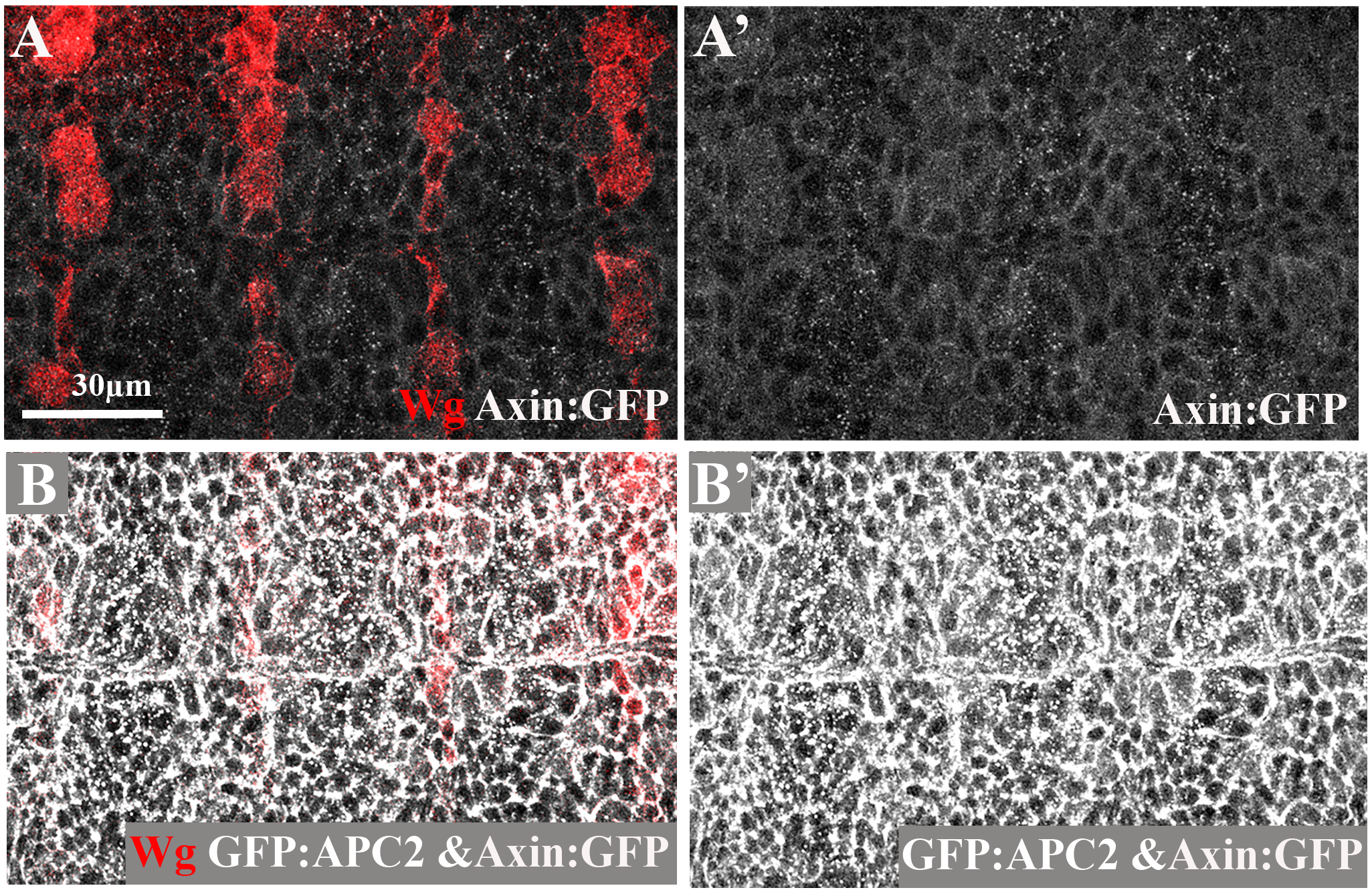

Supplement: S4 Fig — (A,B) Fixed stage 9 embryos imaged under the same microscope conditions, illustrating that when expressed using the MatGAl4 drivers, GFP:APC2 is substantially brighter under our conditions than Axin:GFP, allowing us define GFP:APC2 genotypes by overall GFP fluorescence. (A) GFP brightness of Mat Axin embryo. (B) Example of a MatAPC2 & Axin embryo we scored in the GFP high category. This embryo would also have been scored as Axin high because of the bright puncta. (TIF) [file pgen.1007339.s004.tif]

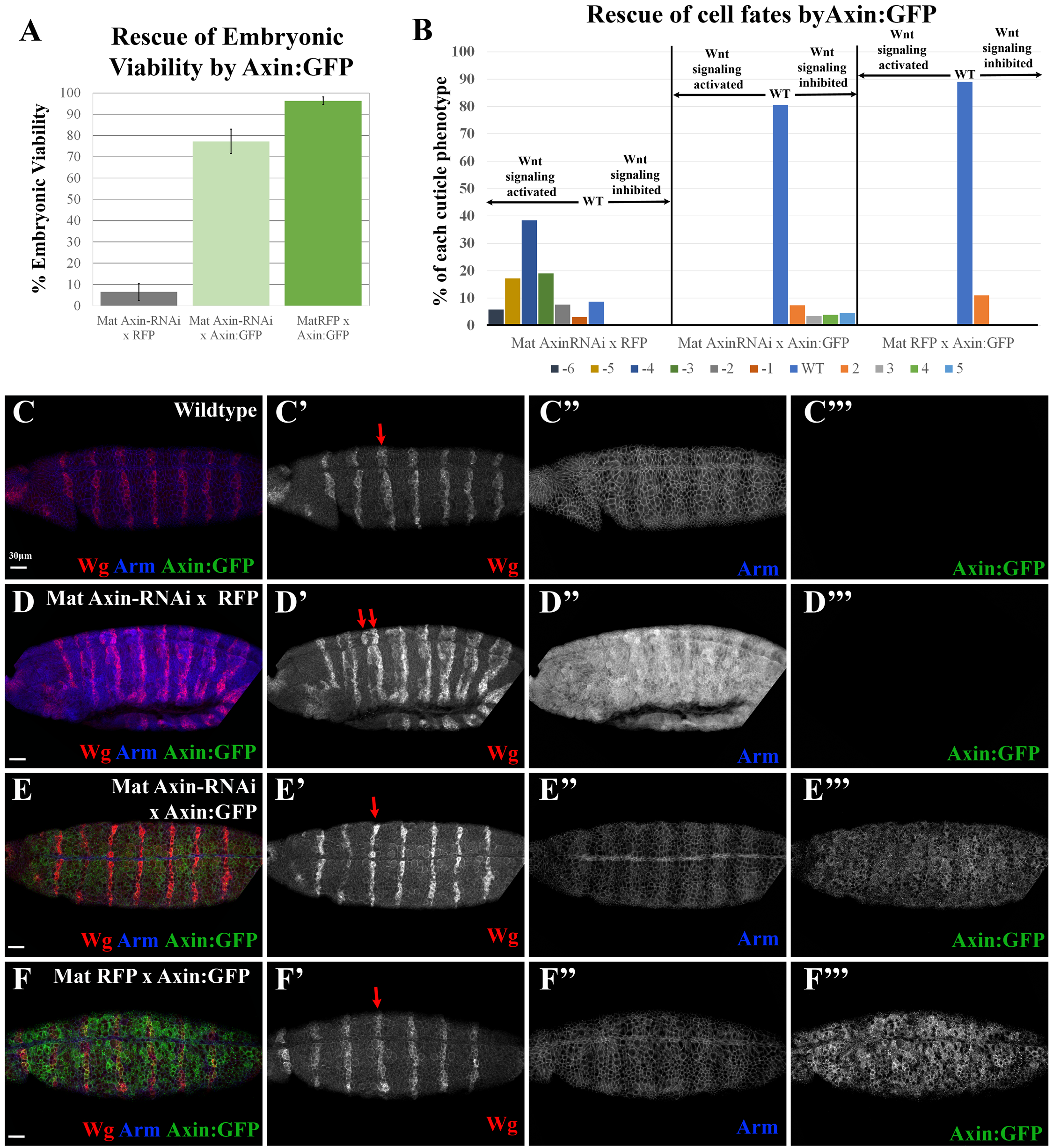

Supplement: S5 Fig — We compared the effects of Axin RNAi with or without expressing Axin:GFP. Crosses: matGAL4/+; matGAL4/Axin shRNA females to either UAS-Axin:GFP males or UAS-RFP males as a control. We also crossed matGAL4/UAS:RFP; matGAL4/+ females to UAS-Axin:GFP males to control for effects of Axin:GFP expression. (A) Assessment of embryonic viability. Axin RNAi leads to highly penetrant embryonic lethality which is largely rescued by expression of Axin:GFP. (B) Assessment of effect on Wg regulated cell fates via cuticle analysis. Categories are illustrated in Fig 3 (reduced Wg signaling) or S8E Fig (increased Wg signaling). Axin-RNAi expands the Wg-promoted naked cuticle fates. This is largely rescued to wildtype by expression of Axin:GFP, though a few embryos lose naked cuticle, as is seen in the control expressing only Axin:GFP. (C-F) Stage 9 embryos, visualize Wg, Arm and Axin:GFP. (C) Wildtype. (D) Axin-RNAi. Note elevated Arm levels and expansion of Wg stripes. (E) Axin-RNAi combined with expression of Axin:GFP. The normal segmental stripes of Arm and the single-cell wide stripes of Wg expression are restored. (F) Expression of Axin:GFP. At this level of expression most embryos have near normal Arm stripes. (TIF) [file pgen.1007339.s005.tif]

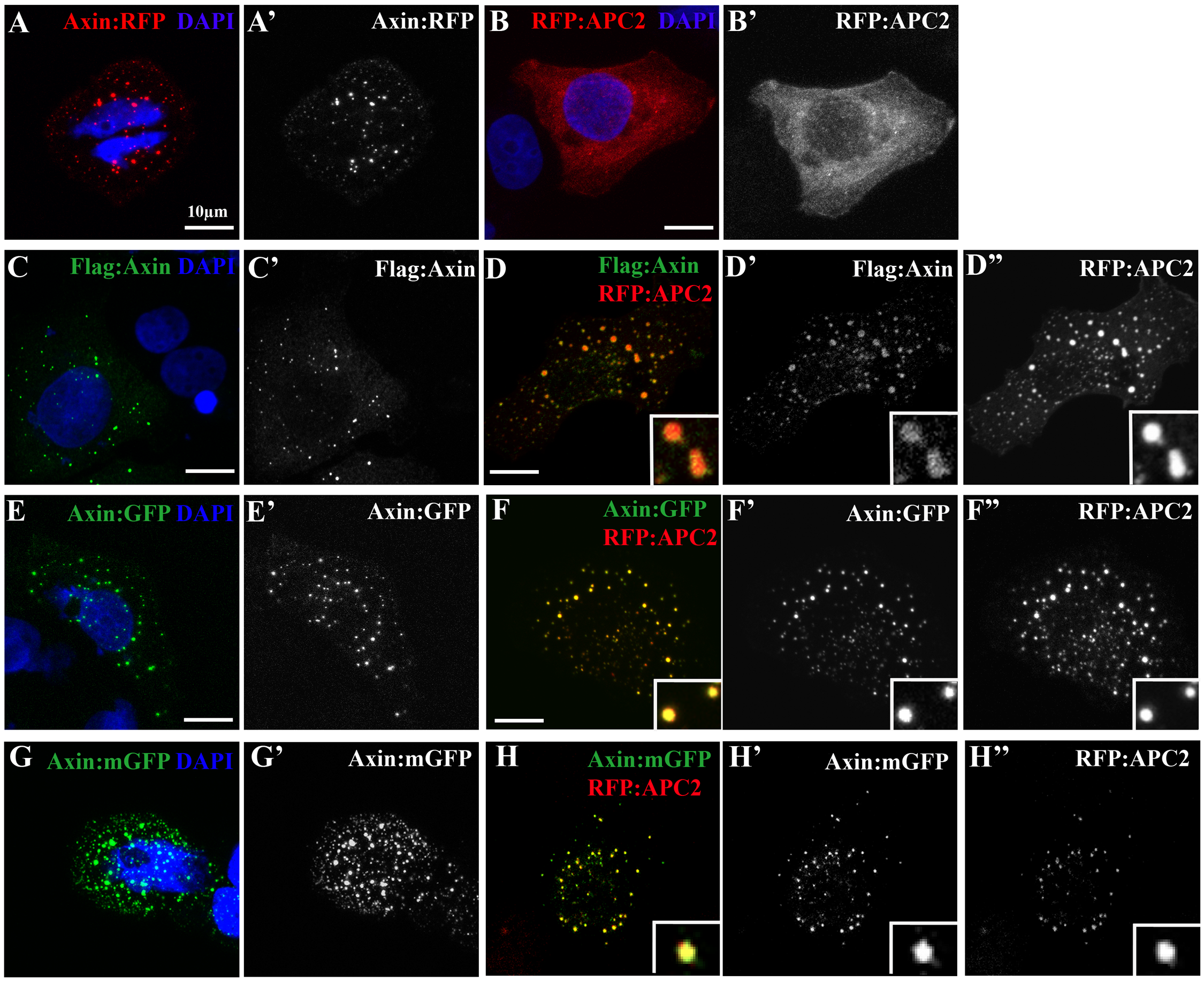

Supplement: S6 Fig — Constructs expressing the indicated proteins were transfected into SW480 cells and visualized either using the fluorescent tag or using an anti-Flag epitope antibody. (A,C,E,G) Axin:RFP (A), Flag:Axin (C), Axin:GFP (E), and Axin:monomeric GFP (mGFP) (G) all assemble into numerous puncta-no differences were seen in this regard (B) RFP:APC2 is diffusely cytoplasmic. (D,F,H) Flag:Axin (D), Axin:GFP (F) and Axin:mGFP (H) can all recruit RFP:APC2 into puncta. Insets = closeups of puncta, illustrating co-localization. (TIF) [file pgen.1007339.s006.tif]

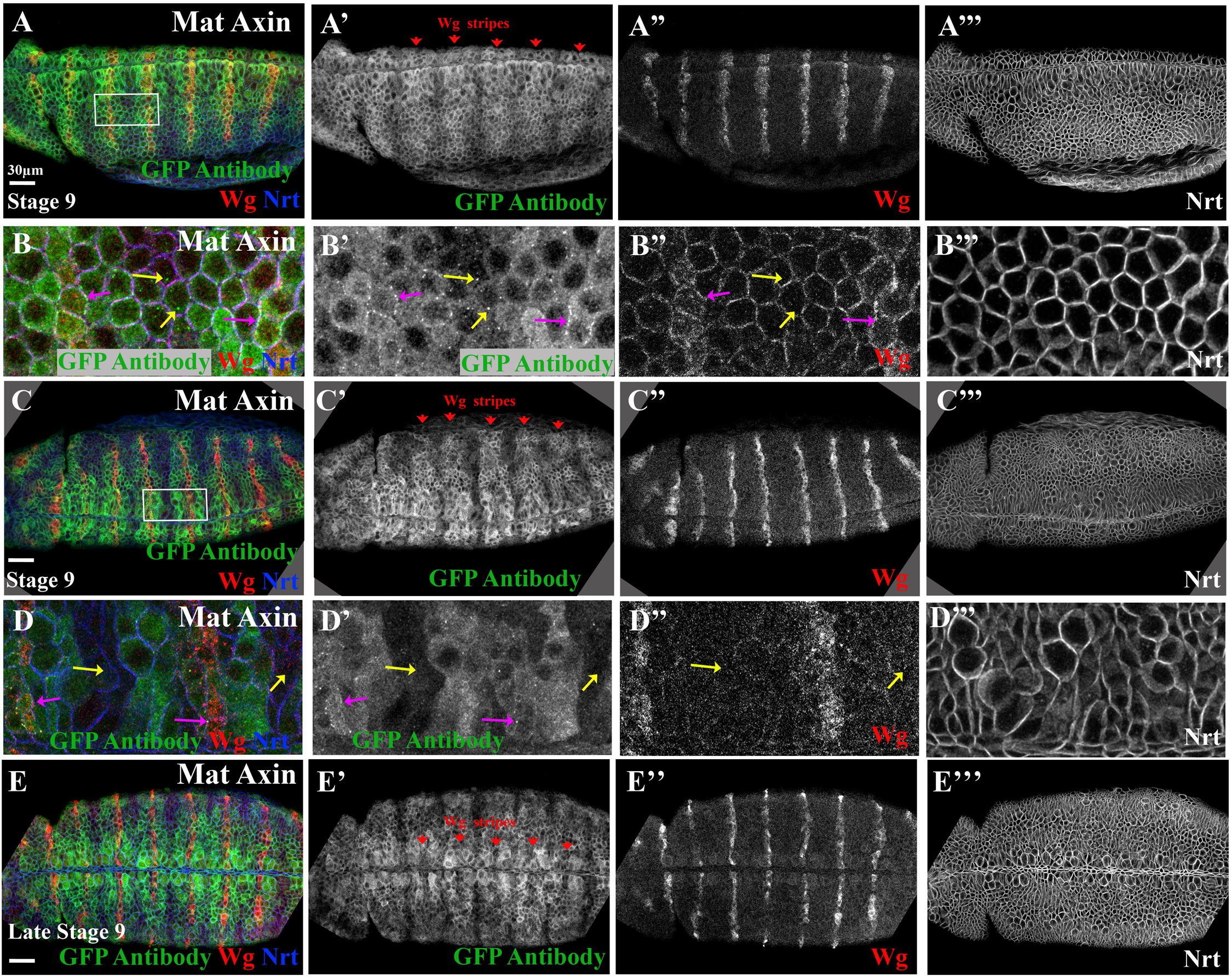

Supplement: S7 Fig — (A-D). Stage 9 embryos, anterior to the left. (E) Late stage 9/stage 10 embryo. All are expressing Axin:GFP using the matGAL4 driver (Mat Axin) and all stained with antibodies to GFP and Wg, along with Neurotactin (Nrt) to visualize the plasma membrane. B and D are close-ups of A and C, respectively. (A,C) Antibody staining clearly reveals elevated cytoplasmic Axin:GFP in cells receiving Wg signal (arrows). (B) In optimally stained embryos, close-ups also reveal both cytoplasmic puncta in Wg-OFF cells (yellow arrows) and membrane-associated puncta in Wg-ON cells (magenta arrows). (D) In many embryos cytoplasmic puncta in Wg-OFF cells are either not visible or less apparent (yellow arrows), while membrane-associated puncta in Wg-ON cells remain visible (magenta arrows) and elevation of cytoplasmic Axin in Wg-ON cells becomes prominent. (E). By late stage 9-early stage 10, we can visualize the decrease in cytoplasmic Axin in cells expressing Wg (arrows), as was previously reported [27]. (TIF) [file pgen.1007339.s007.tif]

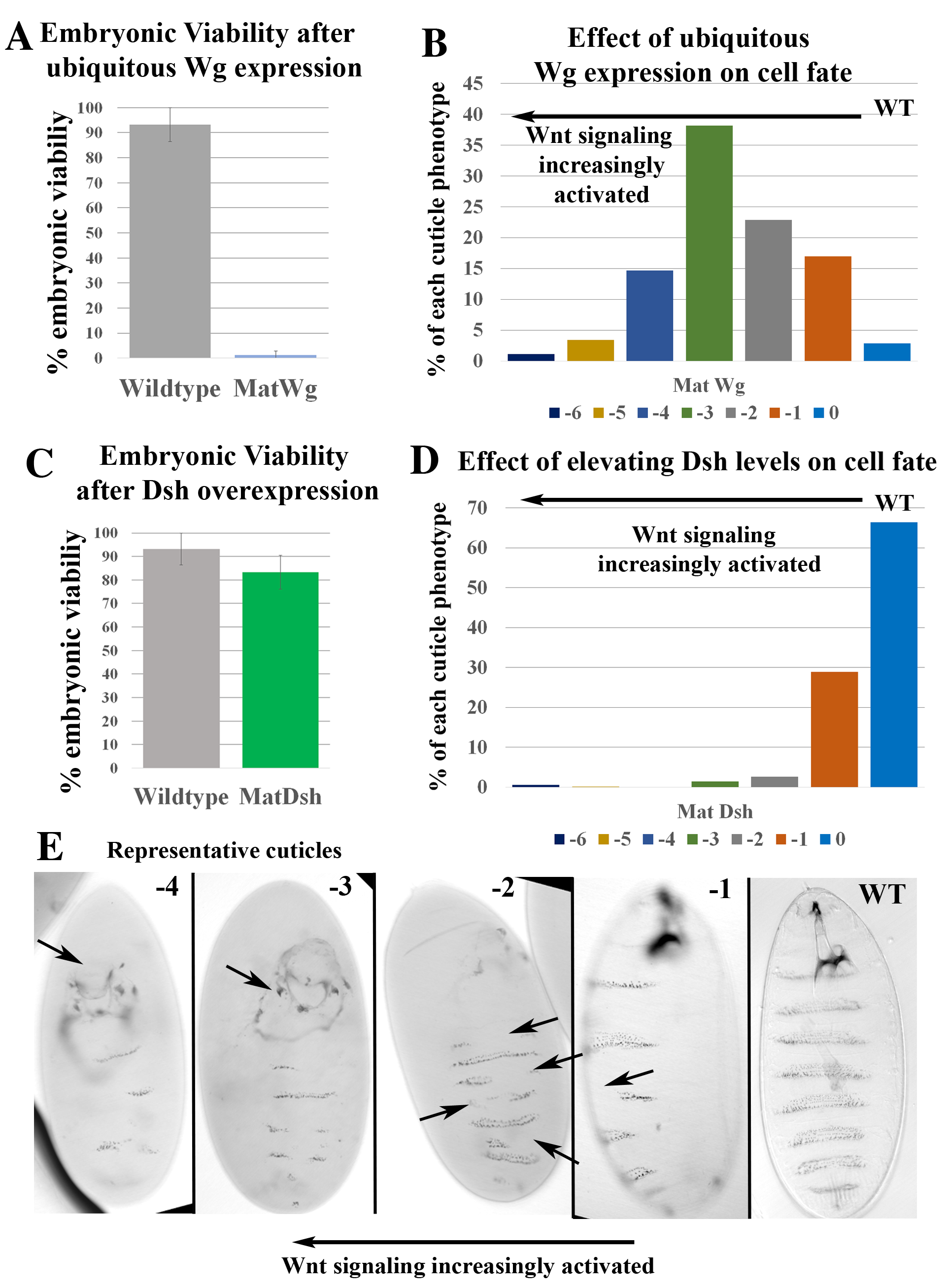

Supplement: S8 Fig — (A) Ubiquitous expression of UAS-Wg:HA using the MatGAL4 driver (Mat Wg) reduces embryonic viability to 1.2%. (B) Ubiquitous Wg expression promotes Wg-regulated cell fates and thus reduces the number of denticle belts formed. (C) Overexpressing UAS-Dsh:Myc using the MatGAL4 driver (Mat Dsh) slightly reduces embryonic viability (83.3 +/- 7.3%). (D) >60% of embryos and larva have a wildtype cuticle phenotype while 29% have a partial loss of denticle belts. (E) Representative images of the phenotypic categories used in B and D. Category 0 = wildtype; Category -1 = At most one denticle belt, or part of 2 belts are absent (arrow); Category -2 = more than half of total denticle belts are present (arrows represent lost denticle belts); Category -3 = less than half of total denticle belts remain, strong head defects or head hole (arrow); Category -4 = A small patch of denticle to none, head hole (arrow). (TIF) [file pgen.1007339.s008.tif]
